# GlyNet: a multi-task neural network for predicting protein–glycan interactions†

**DOI:** 10.1039/d1sc05681f

**Published:** 2022-05-16

**Authors:** Eric J. Carpenter, Shaurya Seth, Noel Yue, Russell Greiner, Ratmir Derda

**Affiliations:** Department of Chemistry, University of Alberta Edmonton Alberta Canada ratmir@ualberta.ca; Department of Computing Science, University of Alberta Edmonton Alberta Canada; Alberta Machine Intelligence Institute (AMII) Edmonton Alberta Canada

## Abstract

Advances in diagnostics, therapeutics, vaccines, transfusion, and organ transplantation build on a fundamental understanding of glycan–protein interactions. To aid this, we developed GlyNet, a model that accurately predicts interactions (relative binding strengths) between mammalian glycans and 352 glycan-binding proteins, many at multiple concentrations. For each glycan input, our model produces 1257 outputs, each representing the relative interaction strength between the input glycan and a particular protein sample. GlyNet learns these continuous values using relative fluorescence units (RFUs) measured on 599 glycans in the Consortium for Functional Glycomics glycan arrays and extrapolates these to RFUs from additional, untested glycans. GlyNet's output of continuous values provides more detailed results than the standard binary classification models. After incorporating a simple threshold to transform such continuous outputs the resulting GlyNet classifier outperforms those standard classifiers. GlyNet is the first multi-output regression model for predicting protein–glycan interactions and serves as an important benchmark, facilitating development of quantitative computational glycobiology.

## Abstract 2

GlyNet, a neural net model of glycan-protein binding strengths. Given a glycan it outputs binding to each of several protein samples. Reproducing glycan array data, it extrapolates the binding of untested glycans against the protein samples.

## Graphical abstract

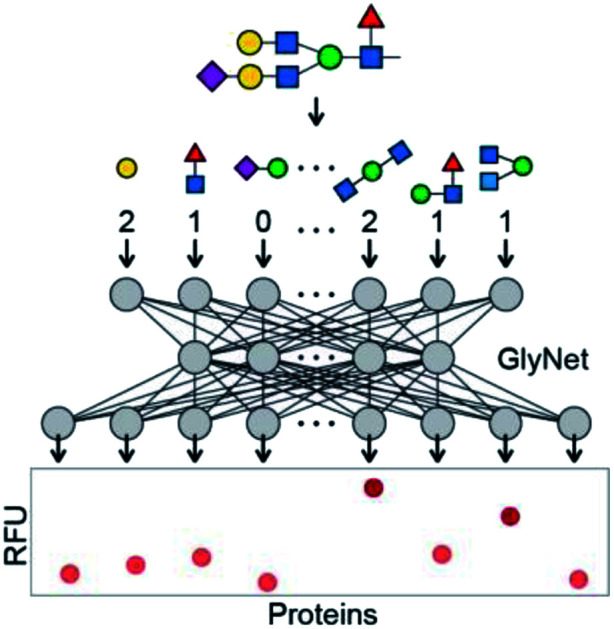

## Introduction

A complex array of carbohydrates (glycans) coat the surfaces of every cell.^1^ Interaction of this glycan coat with protein receptors on human cells is the first step in distinguishing human cells from invading pathogenic bacteria and viruses, or detecting cancerous cells in human tissues.^3^ A major tool for collecting glycomic data, the glycan array, is similar to DNA microarrays:^4^ a variety of carbohydrates are deposited onto distinct locations on a glass surface^5^ or onto distinct beads. Such arrays can simultaneously measure the binding strengths of several hundred carbohydrates with a given sample of a glycan binding protein^6,7^ (GBP), producing a glycan binding profile for that protein. These data are a critical starting point for fundamental applications such as improved designs of inhibitors, vaccines, and therapeutics.^8–13^ Despite the fundamental importance of glycans, their investigation is orders of magnitude slower than the study of proteins or nucleic acids, for several reasons: (i) glycans are not directly encoded by DNA:^1^ thus, “glycomics” information cannot be collected using powerful next-generation sequencing techniques. (ii) As glycans are not linear structures, analytic techniques for linear sequences cannot be readily applied to their graph structures. (iii) There are relatively few glycan oligomers as their laboratory synthesis is significantly more difficult than synthesizing amino acid oligomers (proteins) and RNA/DNA oligomers. Avoiding these difficulties is why it would be valuable to accurately predict the strength of the interaction between any given glycan and protein pair; this requires methods that can effectively extrapolate beyond our limited experimental knowledge of glycomics.

The ability to predict protein–glycan interactions, even approximately, reduces the search space that needs to be explored by experiments. Not only is the space of glycans difficult to explore, it also scales significantly faster than the analogous space of DNA–RNA or proteins. For example, considering glycans composed of just 10 common mammalian monosaccharides with 8 different linkages between them (α2, β2, α3, β3, α4, β4, α6, or β6), there are 56 880 trisaccharides (see ESI Table S1† for a list of these structures). It is not currently feasible to synthesize this set, much less explore their interactions. These are small structures; this glycan count increases to 4.4 × 10^6^ tetrasaccharides (also listed in ESI Table S1†) and 3.8 × 10^8^ pentasaccharides; enlarging the constituents to the dozens of monosaccharides and considering the full linkage varieties, the number of possible glycans grows even larger. Collective human knowledge in glycomics falls several orders of magnitude short. For example, GlyTouCan 3.0, today's most exhaustive database of glycan properties, contains information about only 120 000 entries composed of more than 100 diverse monosaccharides.^14^ To understand bio-logical/biochemical processes it is critical to know whether a specific glycan interacts with a particular protein, and how strongly; datasets that describe glycan–protein interactions contain only a small subset of those glycan structures.^15^ Advances in understanding peptide and nucleic acid sequences is fuelled by tools that give rise to experimental libraries of millions of instances and high-throughput sequencing that can exhaustively analyse them. Unfortunately, state-of-the-art glycan synthesis approaches generate libraries of only hundreds of glycans;^16,17^ and parallel testing of glycan properties on glycan arrays yields information about only hundreds of structures. The fundamental challenges in synthesizing glycans and collecting new glycomics data motivated our work to extrapolate beyond the existing glycan–protein binding data.

Previous machine learning (ML) approaches to protein–glycan interaction employed techniques like support vector machines,^18,19^ graph kernels,^19^ modularity optimization methods,^20^ and Markov models,^21–23^ to identify glycan motifs, substructures that specific proteins recognize – for reviews see Mamitsuka,^24^ Haab,^25^ and Sese.^26^ Several publications from Aoki-Kinoshita and co-workers,^21,23,27–30^ Guy and co-workers,^31^ Cummings and co-workers,^15^ and recently Bojar and co-workers,^32–35^ focus on ML classification models that predict qualitative features (think “strong *vs.* weak” interactions) for each glycan–protein pair. Woods and co-workers combined molecular mechanics, automated 3D glycan structure generation and docking techniques to produce computational carbohydrate grafting that can qualitatively predict the atoms binding carbohydrate fragments with 3D protein structures.^36^ Malik and Ahmad provide a learned model for the reverse task, identifying learned combinations of features of a protein that predict whether it binds to a particular glycan.^37^

In recent years, ML algorithms based on Neural Networks (NN)^38,39^ have achieved remarkable success classifying images, sound, text and linear biological oligomers (proteins, DNA, RNA). Dahl,^40^ Ma,^41^ and Jensen *et al.*^42^ pioneered the use of deep NN to predict physicochemical properties of organic molecules. Neural networks have also been used to predict patterns of enzymatic processing on glycopeptides,^39^ taxonomic classifications of organisms synthesizing glycans,^34^ and biological properties like immunogenicity of glycans.^33,35^ During the preparation of this manuscript, Bojar and co-workers released as a preprint^43^ and then a peer-reviewed^35^ description of the first graph neural network (GNN) that predicts properties of glycan graphs such as evolutionary origin, immunogenicity and recognition by viral proteins. Here we advance the state-of-the-art developing a regression model with quantitative outputs of protein–glycan interaction using neural networks. Since the initial disclosure of the GlyNet architecture as a BioRxiv preprint,^44^ other manuscripts have used it as a benchmark for their tools reporting binding to lectins: LectinOracle^45^ and glyBERT.^46^ GlyNet, thus adds to a series of important benchmarks that drive improvements in ML models for glycobiology.

**Fig. 1:**
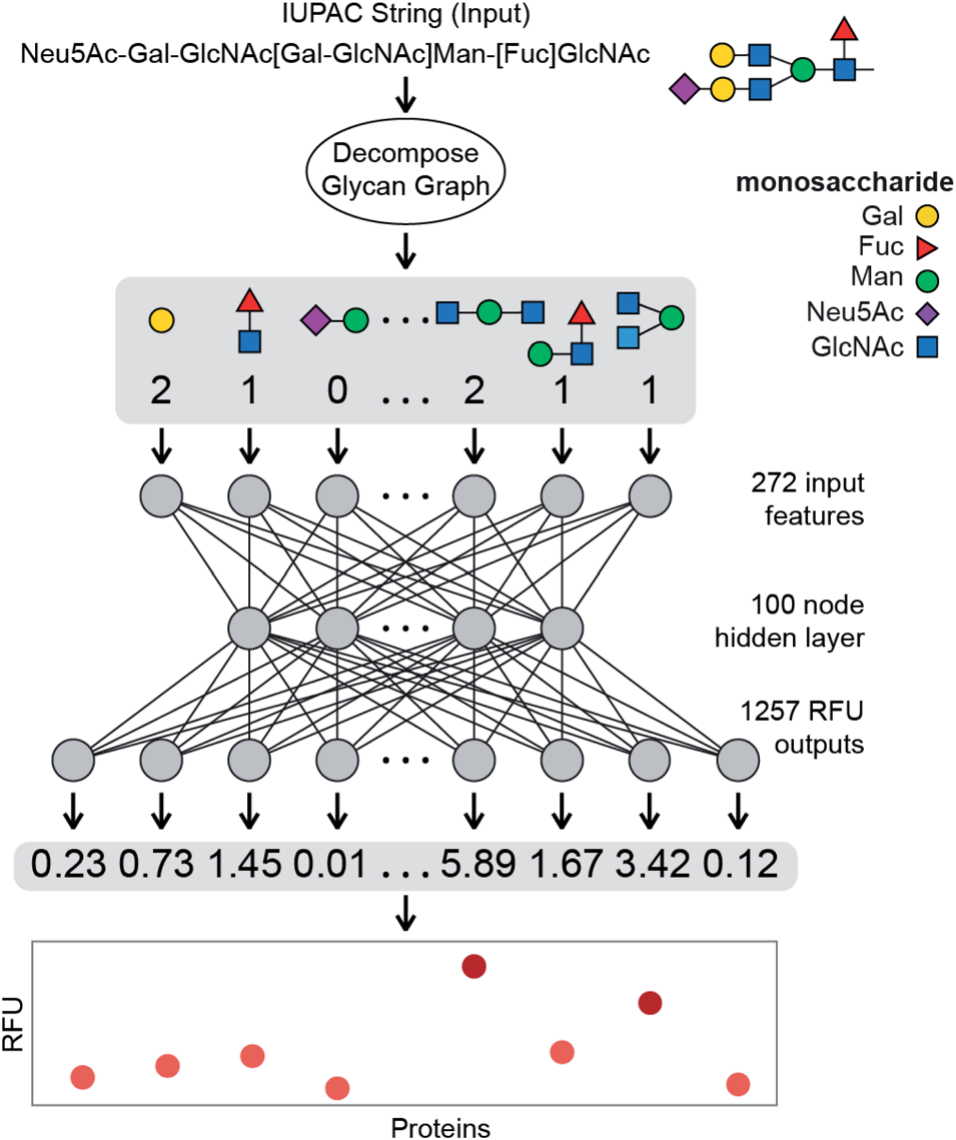
Schematic overview of the learned model described in this paper. Each glycan is decomposed into a fingerprint of feature counts, primarily of small *q*-gram subtrees (here *q* = 1, 2, or 3). This fingerprint is then input into the learned model which outputs the predicted binding value between the glycan and each of the 1257 protein samples.

Binding of a given glycan to a specific protein is a fundamental biological process, and the strength of this interaction—AKA affinity and avidity—is the foundation of most biological responses elicited by glycans. Here, we develop a NN that takes a glycan structure as an input and outputs quantitative binding values, *i.e.* the “strength” of the interaction, for a specific fixed set of proteins. Training a NN typically requires large numbers of labelled instances – here, sets of protein–glycan pairs, each labelled with the associated *K*_d_ (avidity and affinity) values. Unfortunately, there are no such datasets readily available. However, as semi-quantitative estimates of glycan–protein binding strength we use relative fluorescence unit (RFU) values obtained in glycan array experiments conducted by the Consortium for Functional Glycomics (CFG).^5^ Note that our approach allows us to learn models that predict binding behaviours of glycans to generic receptors, based solely on the observed behaviour of glycans included in the CFG arrays. We do not have to know the protein sequences of these receptors; in fact, they do not have to even be proteins. This is an important advantage of our target-agnostic approach over alternative algorithms that require knowing a homogenous sequence (and often a structure) of the target.^35,45^ A target-agnostic approach is commonly used in traditional drug-lead discovery that aims to use existing binding data for a specific target to predict molecular structures that have improved interactions.^47^ In this manuscript, we propose a system in which a glycan is encoded as an input into a neural network, which then predicts the RFU values for that glycan across a large set of protein samples (Fig. 1). We use 752 943 RFU values from the binding of 599 glycans to 353 proteins at different protein concentrations (1257 samples total) to train the neural network, providing a powerful example of a regression model that will predict continuous RFU values for an input glycan across this diverse set of protein samples; here based on a multi-output neural network architecture.

## Results and discussion

### Selection of CFG glycan array protein binding data

**Fig. 2:**
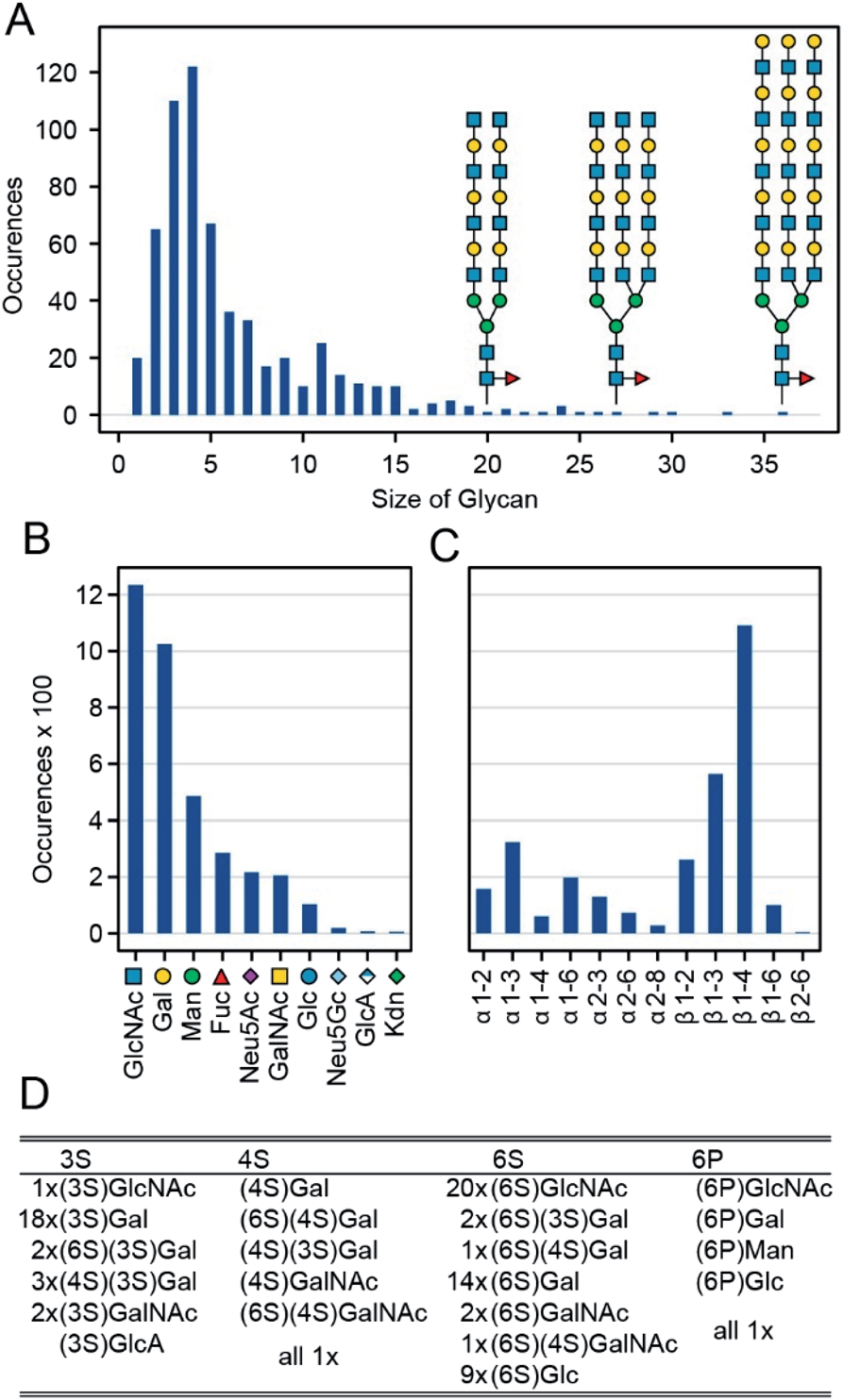
Glycans used for the training of GlyNet. (A) Distribution of the glycan sizes and examples of bi- and tri-antennary glycans with 20, 27 and 36 monosaccharides. (B) Distribution of monosaccharide occurrences within the 599 glycans. (C) Distribution of the different anomeric link occurrences within the glycans. (D) Details of the modified monosaccharides, showing for each of the four modifications/positions a list of the monosaccharides with that modification and their frequencies.

In general, a glycan array contains several dozen to several hundred glycans, each printed on one or more discrete spots; when exposed to a given protein sample, one can then read off the RFU for each spot. Combining the data from many such sample–array pairs provide convenient and uniform data for training and large-scale analysis.^20,26,29,31^ Specifically, we combine data from 1257 such experiments run on CFG's v5.0, v5.1 and v5.2 Mammalian Printed Arrays. We omitted glycan data prior to version 5.0 CFG arrays because these arrays contain fewer glycans then the version 5.0–5.2 arrays. For our initial experiments we only used version 5.0–5.2 arrays giving the simplicity of a uniform dataset with RFU values available for all glycan–protein interactions. We then demonstrate that datasets with missing data can also be used for training in our architecture, but we focus the majority of this manuscript on work with the uniform version 5 data. We have omitted from this dataset two glycans not available on the v5.2 arrays, and two glycans with errors in the reported structure. We also omitted eight glycans containing one of five rare monosaccharides (each appearing four or fewer times among all the glycans: Rha, GlcNGc, G-ol, MurNAc, Neu5, 9Ac2). Specifics of the omissions are in the ESI (Table S2†). The remaining glycans range from 1 to 36 mono-saccharides in size (mean = 6.1, median = 4, Fig. 2A), they collectively contain 10 different monomers and 12 anomeric linkages (Fig. 2B). ESI Table S2† lists the 599 glycans as well as the omitted ones (Fig. A3). In addition to the ten main monosaccharides, there are also phosphorylated or sulfurated units, listed in Fig. 2D.

**Fig. 3:**
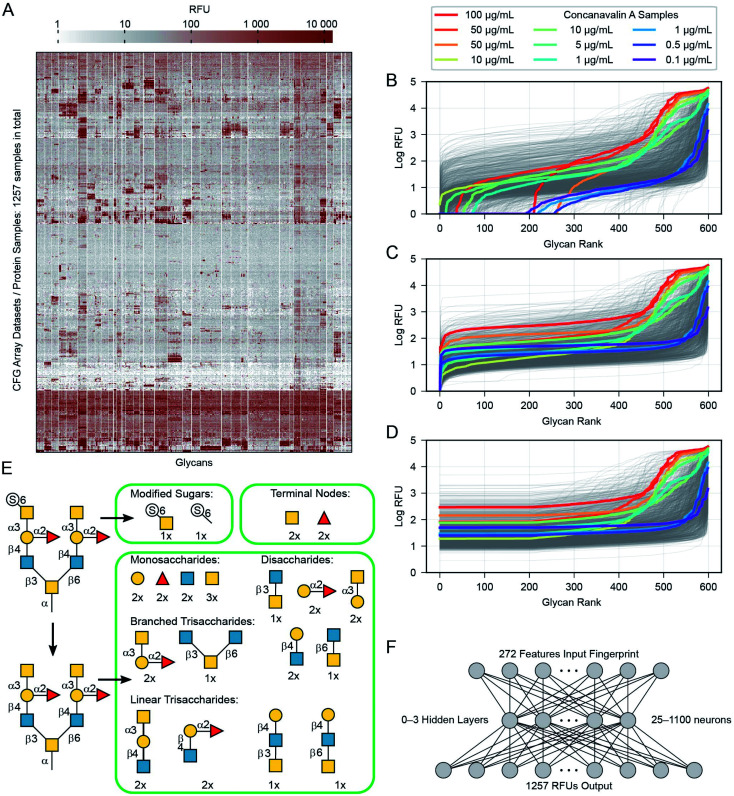
Pre-processing of data. (A) Heatmap of the data overall, with all glycans and protein samples, each row and column has been clustered, appearing next to similar rows and columns. (B) The 599 mean RFU values for each protein are sorted and plotted as a line. Most of the 1257 proteins appear as a grey line, but several samples of the protein concanavalin A at various concentrations are highlighted in colour (due to the sorting, glycans appear at different *x*-axis positions for each protein). (C) The raw values are incremented as needed to place the minimum at 1.0. (D) The lowest 1/3rd (200 values) are replaced with the 200th value to reduce noise in the training data. (E) Details of the subgraph features used to describe each glycan. (F) Diagram illustrating the neural network architectures we used in this work. One hidden layer is shown, although we experimented with networks that contained zero to three hidden layers.

We extracted raw data from a table in the ImaGene^48^ program's format. This table contains full details of the fluorescence measurements, including signal and background values for each spot. Not every CFG dataset includes this table, we include all 1257 version 5 samples with the detailed ImaGene data, and other than this restriction we did not censor any samples or remove samples from the CFG database. Every sample submitted to CFG has a value, it passed the internal review and the array experiment was performed for a reason. The earlier researchers who constructed the CFG dataset had reasons to consider each sample to be important. ESI Tables S2 and S3† provide a full list of the protein samples and glycans including their associated CFG labels. Fig. 3A shows all of the data as a heatmap, similar to analyses by Sese,^26^ a larger version of this panel with labels is available as ESI Fig. S1.† Overall, 881 of the protein samples correspond to 352 unique proteins (cpbIds); the remaining 376 are complex samples (serum, *etc.*). Notably, we are able to model the behaviours of these complex samples because we do not need to know anything about the composition the sample in order to build our models. Many of the protein samples were tested at several different concentrations (coloured lines highlight Concanavalin A at various concentrations in Fig. 3B–D). Specifically, we include binding values of 18 proteins measured at 5 or more different concentrations and 69 other proteins measured at 4 distinct concentrations (ESI Table S3†). Since such concentration scan data are relatively rare in the CFG dataset and concentrations are not specified for ∼120 samples, we treat each protein-concentration pair as a unique sample and consider its binding with respect to the 599 glycans.

### Pre-processing of glycan–protein binding data

Our pre-processing method combines ideas from Guy and coworkers^31^ and our general knowledge about the nature of the RFU data. For each glycan–protein pair, the glycan arrays measure Relative Fluorescence Units (RFUs), which are a measure of the binding strength for that pair. As each glycan occurs six times in the array, we use the mean over these six replicate array spots for each glycan. We modify these input values in three steps: (i) we add a constant to all RFU values for each glycan array to set the minimum value in each protein-profile to 1.0 (Fig. 3B) and (ii) log-transformed the values (Fig. 3C) to reduce the several orders-of-magnitude of dynamic range (both similar to a previous report^31^). (iii) We then replaced the lowest 1/3rd of these values (data for 200 of 599 glycans) with the value equal to that of the 200th lowest point in that dataset (Fig. 3D). This transformation eliminates the noise/variability at the lower end of the log-transformed dataset caused by fluctuations in the readout machinery, wash conditions and other factors not relevant to the task of learning glycan–protein interactions. Note that the weakest RFUs are non-binders, and we observed that the lowest third contains the worst noise. “Filtering” more glycans risks removing data about actual glycan–protein binding. The filtering is intended to simplify the learning task, as the model learned from the training process does not need to reproduce any of the irrelevant patterns in this part of the data. The pre-processed log-mean RFU values are available in ESI Table S4.†

### Representation of glycans for input to the neural networks

In order to input a glycan structure into the neural network, we adapted the *q*-gram^49^ approach, encoding each glycan as a “fingerprint”, *i.e.*, a feature vector containing counts of how often each feature occurs. The major features we included were the contiguous 1, 2, and 3-monosaccharide subgraphs of the glycan structure including the connecting anomeric linkages (Fig. 3E). Including the smaller subgraphs allows the 85 mono- and di-saccharides to be represented (14% of the glycans), as they have no tri-saccharide subgraphs. Our feature set also included counts of the terminal monosaccharides, those at the non-reducing ends of the glycan, and the most exposed in protein–glycan interactions. To deal with phosphorylation and sulfation of glycans: we used the unmodified sugar in the structure used for subgraph generation and terminal positions, but also added features for the phosphate or sulfate group position, and a feature for each such modified monosaccharide – see Fig. 3E. This encoding helps to avoid a combinatorial explosion in the subgraphs created by these variants.

The final feature vector for each glycan contains one element for each feature (272) found in any of the 599 glycans. The whole of the training set is thus a set of 599 instances, each of which has 272 features describing the relevant glycan (used as model inputs, available in ESI Table S5†) and 1257 RFU values (target model outputs, available in ESI Table S4†), one for each protein sample.

This fingerprint encoding does not describe everything about the glycan, in particular it does not encode the location of a feature (substructure), only how often it occurs. Two (or more) distinct glycans may have identical feature vectors and are thus treated as identical. In the set of 599 glycans, we have three cases in which the same feature vectors encoding multiple glycan structures (ESI Fig. S2†). Also, for 105 of the 599 glycan instances the CFG description does not record whether the anchoring monosaccharide is in the alpha or beta conformation (ESI Table S2†). To avoid introducing additional complexity with this ambiguity, we ignore the stereochemistry of the anomeric carbon of this position in all cases and similarly, we do not model the CFG spacers and linkers^50^ nor even encode which spacer (ESI Table S6†) links the glycan to the substrate. The consequence of these design decisions is an inability of the network to distinguish glycans that may exhibit different binding properties. For example, the binding of Gal(β1–3)GalNAc to Fm1D has more than two-orders of magnitude difference across the selection of α–Sp8, α–Sp14, α–Sp16, and β–Sp8 for the anomeric stereochemistry and the linker structures present in the CFG data (ESI Tables S2 and S4†). When we train the network, all four of these are distinct input instances and the model output is evaluated with respect to each of the corresponding outputs. As the model produces the same output for all these indistinguishable inputs, the prediction can never exactly match, but will be optimized towards a compromise, which here means returning the means of the CFG values. However, these instances are not conflated with the Gal(α1–3)GalNAc ones because the stereochemistry of all other subunits is input to the network. Our encoding assigns 599 CFG glycans to 515 distinguishable cases; while there are other encodings that avoid such indistinguishability of glycans,^35,45,46^ our learned model may not be able to extract patterns of glycan–protein binding from them. Despite the obvious limitation of indistinguishable glycans, we believe our system achieves sufficient accuracy to be useful.

### Multi-task network

**Fig. 4:**
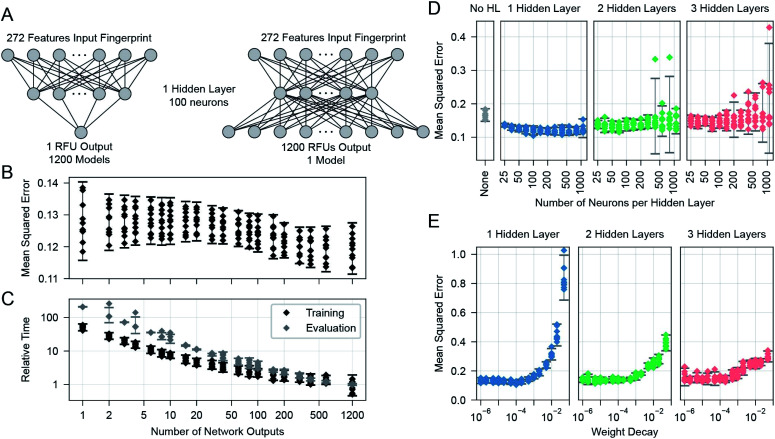
Searches were used to find optimal parameters and settings. (A) Effect of varying the number of outputs of the neural networks, from one (corresponding to a single protein sample), to 1200, corresponding to this number of protein samples. (B) The mean-squared error, as a function of the number of outputs. Multiple models with each output size were trained to evaluate the same set of 1200 protein samples for all output size cases. (C) Overall relative training and evaluation times to produce the 1200 outputs. Evaluation time points are for a single evaluation of the models on all 599 glycans (producing one 599 glycan × 1257 protein sample output set) and are often overlapping in the plot except for occasional outliers. (D) The cross-validated MSE effects of altering the number of hidden layers and their number of neurons. (E) Effect of varying the weight decay parameter of the ADAM optimiser. In (B) to (E): each error bar is from *n* = 10 points, one from each of the 10 folds of a single run across 599 glycans. Error bars show the 95% confidence interval for a Gaussian of the same mean and standard deviation as the plotted points.

We learned the final multi-task model, named GlyNet, from all the CFG data. To find the best model the learner used *k* = 10-fold internal cross-validation (CV) to identify the appropriate number of hidden layers, the number of nodes in each hidden layer, as well as other hyperparameter settings (Fig. 4). We randomly partitioned the glycan samples into ten folds, trained a model using the glycans in nine of the folds and validated it on the tenth fold using Mean Squared Error (MSE) as the loss function. We ensured that glycans encoded by the same fingerprints (see above) are partitioned into the same fold. By repeating this process, building separate models holding-out each of the ten folds, we obtained the mean performance of our architecture on the data. Our optimization used ADAM^51^ implemented in the PyTorch^52^ ML library, with batches of 64 instances; we stopped training after 1000 epochs, or early if the MSE did not improve for 10 epochs.

There are many ways models can predict protein–glycan interaction values. We could construct one learned model for each protein: given a description of a glycan, it produces the RFU of this protein with the given glycan. Alternatively, a multi-task approach, adapted from Dahl *et al.*:^40,41^ given a glycan, the model simultaneously outputs RFU values for each of the 1257 proteins, with this glycan – see Fig. 1. This means training only one model, rather than 1257, which greatly reduces overall training time and allows for a more extensive hyperparameter search. More importantly, we found that increasing the number of output neurons improved the performance of the model (Fig. 4A). The multi-output network has a better mean squared error (over all 599 training glycans) than a single output network on 75% of the protein/concentration pairs, with a median improvement in the MSE of 10%. These results led us to focus on models that, given an input glycan, simultaneously output all 1257 (logarithmically transformed) RFU predictions. As shown in Fig. 3F, our neural network architecture first translates the input glycan into intermediate “hidden layers”, which are then used to generate the (log scaled) RFU values for the 1257 protein-concentration instances. For this to work, the hidden layers need to implicitly represent the glycan characteristics that determine the RFU values for all the proteins. Note that each training instance (one glycan and 1257 RFUs) provides a great deal of feedback (1257 values) for each of the “intermediate” weights, which suggests it may be relatively easy to train this structure compared to the single output approach. Moreover, we know that there are general principles that guide protein–glycan interaction,^53^ which we believe intermediate layer(s) may (implicitly) encode. We also explored intermediate cases, where multi-output networks produced RFU values for only some of the protein samples. We found no improvements when each multi-task predictor produced under ∼10 outputs, then saw gradual improvements as the number of outputs increased (Fig. 4B). We also found that the overall training and evaluation times decreased with greater numbers of outputs (Fig. 4C).

### Network training

We found a single hidden-layer of 100 nodes optimized with a weight decay of 10^−4^ to be the best as assessed by internal cross-validated MSE. In similar learning tasks, Dahl^40^ and Ma *et al.*^41^ previously reported that a multilayer architecture could yield a better performance than a single layer. It is possible that with more data, a deeper architecture could yield a better performance; however, given the rather limited dataset, we proceeded with our simpler single-hidden-layer architecture.

We used neurons with biased ReLU activations for all layers except the final layer, which uses biased linear activations instead. Initial versions of the multi-task networks with ReLU in the final layer trained sub-optimally, with a small number (∼20) of the RFU outputs predicting zeros for all input glycans. These outputs had become trapped in a zero-gradient state and were not modified afterwards by the learning process. This problem was avoided after we switched to linear output neurons, which have a non-zero gradient everywhere.

### Evaluation of the glycan binding model by glycan

After selecting the optimal architecture and producing models using the 9/10ths of the training glycans, we compared outputs on the held-out tenth folds, with true RFU data obtained from the CFG arrays. Over all 599 glycans for each of the 1257 proteins we achieved a cross-validated mean-squared error (MSE) of 0.12 and mean *R*^2^ = 0.78 (coefficient of determination – a measure of correlation) in predicting logarithmically transformed RFU values.

**Fig. 5:**
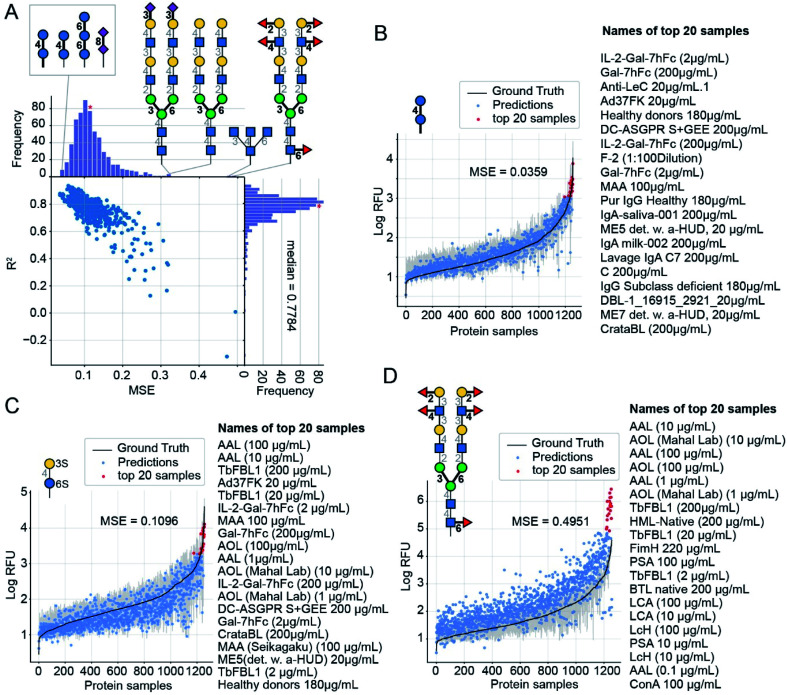
GlyNet predictions across the 1257 protein samples for specific glycan inputs. (A) Plot of MSE *vs. R*^2^ values for 599 GlyNet predictions that describe binding of individual glycans to 1257 protein samples. Each dot represents MSE and *R*^2^ values from predictions at one output for 599 glycan inputs. Also shown are the structures of the glycans with the lowest MSE (best accuracy) and highest MSE (worst accuracy). (B–D) GlyNet predictions for three glycan inputs: the lowest MSE, a median MSE and the highest MSE. In (B)–(D) dots are RFU values predicted by GlyNet and the ground truth CFG values are plotted as a solid black line with a grey 95% CI band, from 6 replicates. The *x*-axis positions in each plot are sorted in the order of increasing ground truth RFU. Red dots highlight the 20 protein samples with the highest predicted RFUs. These twenty samples are listed (highest to lowest) to the right of each plot. The glycans are (B) Glc(α1–4)Glc(α–Sp8) (https://youtu.be/biWNApZHMP8?t=0) (C) (3S)Gal(β1–4)[Fuc(α1–3)](6S)Glc(–Sp0) (https://youtu.be/biWNApZHMP8?t=303) and (D) Fuc(α1–2)Gal(β1–3)[Fuc(α1–4)]GlcNAc(β1–2)Man(α1–6)[Fuc(α1–2)Gal(β1–3)[Fuc(α1–4)]GlcNAc(β1–2)Man(α1–3)]Man(β1–4)GlcNAc(β1–4)[Fuc(α1–6)]GlcNAc(β1–4)[Fuc(α1–6)]GlcNAc(β–Sp19) (https://youtu.be/biWNApZHMP8?t=598).

For each glycan input, our model outputs its RFU predictions for each of the 1257 protein samples (available in ESI Table S7†). Knowing the “true” values, we can compare them to those predicted values to obtain MSE for this individual glycan. Using 10-fold CV, we found these MSE values vary by more than an order of magnitude, from 0.036 to 0.495; Fig. 5 compares predictions for the glycans with the lowest MSE = 0.036 (Fig. 5B), a median MSE (Fig. 5C), and the highest MSE = 0.495 (Fig. 5D). Predictions are denoted with dots and the ground truth, log(mean RFU), is depicted as a solid line with a 95% confidence interval (grey band, calculated from the six experimental replicates of the RFU data). While we do not use the confidence interval, neither for training nor for calculation of MSE, note that many predictions reside within the experimentally determined confidence interval of RFU values. In other words, the model performance, while not perfect, is comparable to the experimental variance of the data. Each plot has a list of the protein samples with the highest 20 predicted RFU values. These top-20 predictions further highlight that the model predictions accurately reproduce the CFG data. On average 11 of the actual top-20 proteins were in the model's top-20 predictions (95% CI = 6–15). For reference, selecting 20 of the 1257 samples at random would match 11 of the top-20 samples only 10–55% of the time (see ESI† for calculations). The top-20 predictions included the protein with the highest RFU value for 78% of the glycans. Full lists of the top-20 predictions are in ESI Table S8.† A weak correlation (Pearson product-moment correlation coefficient: −0.4) between the number of entries common to the top-20 lists and the MSE of the glycan indicates that even in samples with poor MSE (*e.g.*, Fig. 5D) our model can effectively predict the top-20 glycans.

For further evaluation, plots of the predictions for all 599 CFG glycan instances are available in the ESI. These are merged in an animation (https://youtu.be/biWNApZHMP8) stepping through the glycans (one per second) by ascending MSE of the glycan over the 1257 outputs. Specific glycans can be accessed by time (see legend of Fig. 5, ESI Table S8† and the legend of the YouTube video).

### Evaluation of the glycan binding model by protein sample

**Fig. 6:**
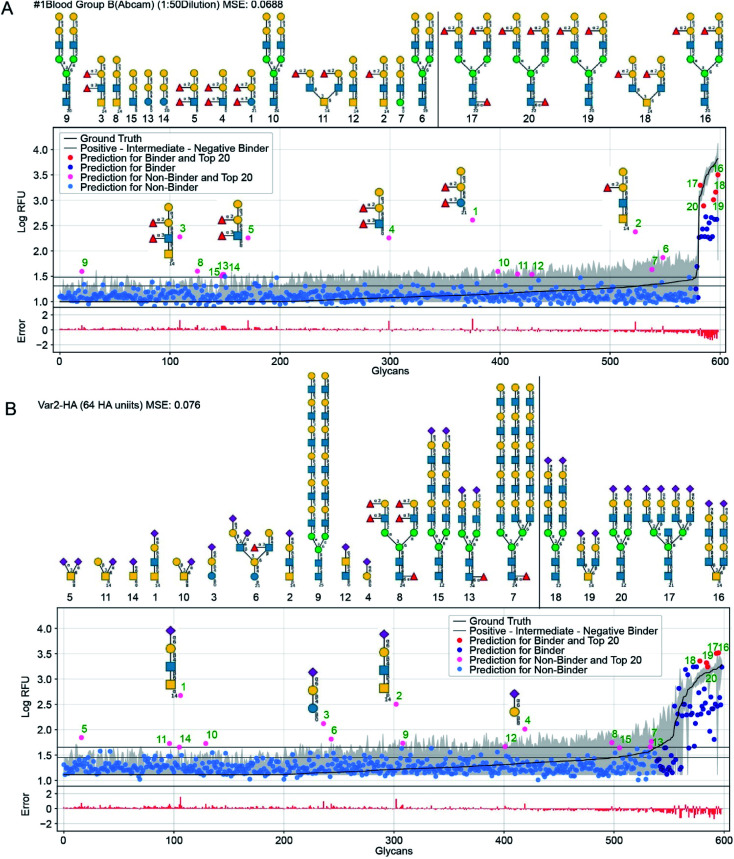
GlyNet predictions across the 599 glycans for two protein samples with well understand recognition profiles. The false positives in GlyNet's learned outputs contain the same iconic motifs as the true positives. The glycans with the strongest predicted RFUs, but which are not binders (by the CFG data) are shown with pink dots (left side), and their structures are shown above the plots. Those with the strongest RFUs and are positive binders in the CFG data are red (in the upper right). Also shown is the mean CFG data (black line) and its 95% CI (grey band) from *N* = 6 replicates. The small numbers at the bottom of the glycan structures represent the spacer/linkers as indicated on the CFG website (https://www.functionalglycomics.org/static/consortium/resources/resourcecoreh8.shtml), and details can be found in ESI Table S6.† (a) Both true and false positives for an anti-blood group B anti-body (https://youtu.be/oHaFF4A22D8?t=374) contain blood group B trisaccharide (Gal(α1–3)[Fuc(α1–2)]Gal(β1–)). (b) A sample of influenza hemagglutinin shows several positive predictions containing Neu5Ac(α2–6)Gal(β1–) (https://youtu.be/oHaFF4A22D8?t=435).

Another way to assess the predictions made by GlyNet is to choose a single output from the 1257 protein-concentration outputs and study it over all 599 glycan inputs. As in the previous discussion, all 1257 predictions are available in several formats: (i) as numbers in ESI Table S7†; (ii) as plots, two examples of which are shown in Fig. 6; and (iii) as a video (https://youtu.be/oHaFF4A22D8) indexed by protein sample in ESI Table S9.†

Fig. 6 shows predictions, focusing on two proteins that have well-understood glycan recognition profiles: an anti-blood group B antibody, and influenza hemagglutinin. To simplify interpretation of the data, we adopt the threshold for positive binders in Coff *et al.*^31^ (median absolute deviation based *M*-score ≥ 3.5) and treat the others (non- and intermediate-binders) as negative binders. Here we focus on false-positives from the GlyNet model. These are the glycan–protein pairs that were not detected as positive-binders in the CFG array data, but which GlyNet predicts to be. We find that many of these false-positives include well-known recognition motifs present in the true positives: *e.g.* GlyNet predicts that many structures with 1-2-linked fucose bind to the anti-blood group B antibody, and many glycans with 2–6 linked sialic acid (Neu5Ac(α2–6)Gal(β1–)) are recognized by influenza hemagglutinin (Fig. 6). To show the generality of the observation, the aforementioned YouTube animation steps through all 1257 outputs (by ascending MSE) and true- and false-positives are shown with red and pink dots respectively. In these, we found that many of the false-positive predictions made by GlyNet have structural similarities with accepted true-positives.

GlyNet maps each of the 1257 protein-concentration instances to a 599-tuple: the RFU values of its binding to the 599 glycans; see Fig. 6. Each can be characterized with its own MSE and *R*^2^ value. The population median of the MSE of these predictions is 0.099 (lower than MSE = 0.11 in Fig. 5A) but we note two major differences: the MSE for individual samples have a wider dynamic range, from 0.007 for a peanut agglutinin sample (PNA 1 μg mL^−1^) to 0.624 for a bacterial sample (HA70 complex-Alexa 500 μg mL^−1^). Unlike our analysis of binding predictions between 1257 protein samples and one glycan, this variant that examines binding predictions between 599 glycans and one protein sample exhibits no obvious correlation between MSE and *R*^2^ (ESI Fig. S3†). To understand this observation, we examined the relationship between MSE, *R*^2^ and the number of glycans that bind to a protein sample. The latter can be defined either as (i) the number of glycans with RFUs that are factor of ten above the “background” RFU (ESI Fig. S4a†) or as (ii) the number of glycans above a threshold for positive binders (based on *M*-score,^31^ ESI Fig. S4b and c†).

When a protein sample does not bind to any glycans, GlyNet yielded the lowest MSEs (ESI Fig. S5†). While accurate, this outcome is not interesting: the model learns that the output is low for every input glycan. It yields a low-signal near-random output with only small differences from the reference data (see ESI Fig. S5† or refer to the first 20 seconds of https://youtu.be/oHaFF4A22D8). The *R*^2^, a measure of correlation between prediction and ground truth, for these samples is low because there is no correlation between the weak signal of the ground truth and the relatively random output of the model (ESI Fig. S5†). This happens because MSE is the loss function optimized during the learning process whereas *R*^2^ was not evaluated or controlled. As the number of strongly binding glycans increases, the MSE of the prediction increases (*i.e.*, absolute error of the prediction increases); at the same time, the *R*^2^ also increases (relative quality of prediction also increases). The 20 to 50 protein samples with the highest MSE values bind to many glycans (*e.g.*, ESI Fig. S3d† or https://youtu.be/oHaFF4A22D8?t=1187 and onwards). They are the most challenging for GlyNet to learn. Notably, the single-output models are no better on these samples, yielding similar performance (ESI Fig. S6†). While these few samples are outliers in the CFG data, it would be interesting to find other learning architectures and/or encoding methods that can improve predictions for these samples.

Another way to study predictions from the GlyNet architecture is to compare data measured on arrays of different composition. While we have focused on data from CFG's version 5 Mammalian Printed Arrays, we extended our model adding outputs from version 2–4 arrays which have fewer glycans than version 5 arrays (more details are given in the ESI,† “Models on expanded dataset” section). Several lectins such as ConA, SNA, PNA, RCA, and UEA I have been measured using both version 5 and earlier versions. For these lectins, we trained GlyNet using log-RFU values from the older arrays and extrapolated the binding of these lectins to the glycans that are absent from the older arrays and evaluated the predictions by comparing them to measurements from similar samples. We observed that the log-RFU values extrapolated from the smaller arrays largely reproduce those measured on version 5 arrays (see ESI Fig. S8–12†). The glycans predicted to have the strongest binding match both the experimentally observed results. Further we see these glycans possess binding motifs previously reported by Mahal and coworkers^54^ suggesting that GlyNet recognizes the same features that have been previously identified as binding motifs for these proteins. The result is that GlyNet makes it possible to compare data from arrays that have mismatched composition by extrapolating the binding for glycans that are not included in all of the arrays. Although we demonstrate this comparison only between versions of CFG's arrays, we expect this approach to also work between data from different array platforms.

### Evaluation of novel predictions made by GlyNet's glycan binding model

Perhaps the most valuable use of GlyNet is to extend the CFG data to additional, untested glycans. We anticipate that this will aid future experiment design by identifying additional glycan structures that are likely to interact with a protein of interest, or given a specific glycan structure, identify proteins it is likely to interact with. As an example, we used the trained GlyNet model to estimate RFU values for 4160 additional glycans, reported to have been previously synthesized or isolated and deposited in the GlyTouCan depository. A recent publication by Agirre and co-workers^55^ highlighted that the GlyTouCan database contains errors in some deposited glycans. We pre-curated the 4160 GlyTouCan glycans to ensure that they contain only the monomers and linkages that can be represented in the CFG training set. In principle, it might be possible to perform a secondary curation of the 4160 structures to ensure that each glycan corresponds either to a previously reported natural or chemically-synthesized glycan. Such curation is an onerous task that extends beyond the scope of this report. We note that the presence of plausible but not yet observed glycans is not a problem *per se* because the ultimate goal of trained models like GlyNet or others^45,46^ is to predict the properties of any plausible glycan (even if such glycan has not been synthesized or isolated yet). We appreciate that the curation of datasets like GlyTouCan is challenging and there is no doubt that the quality of these glycan datasets will improve in the future. As errors in the deposited structures are corrected, GlyNet can be used to generate new predictions for the corrected structures.

**Fig. 7:**
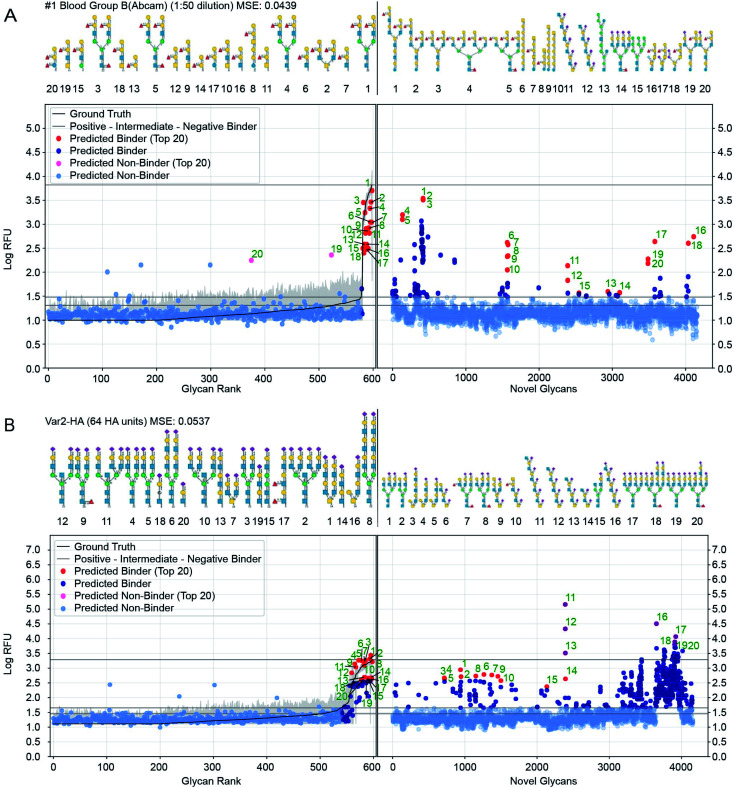
Prediction of glycan binding of Blood Group B (1 : 50 dilution) and Influenza Var-2-Hemagglutinin to a set of 4160 novel glycans. On the left of the plots are the GlyNet predictions across the 599 CFG glycans highlighting (in red) the 20 glycans with the highest log-RFU values (strongest binding), with structures shown above the plots. On the right are predictions of glycan binding on the set of 4160 novel glycans (ordered alphabetically), similarly highlighting 20 strongly binding glycans from across the set and drawing their structures above the plots. Also shown is the reference mean CFG data (black line) and its 95% CI (grey line) with *N* = 6. Glycan structures drawn with DrawGlycan-SNFG.^2^

Fig. 7 shows further details of the predicted responses of these glycans with each of 1257 CFG samples (*i.e.* 352 specific proteins at various concentrations from 376 unique samples). Animations scanning through all the output proteins are available (https://youtu.be/468Rj9ynDW4 alphabetically by protein, and https://youtu.be/pa_6nO0Zl64 clustered by similar RFU patterns). The RFU predictions used in these plots and an index of times by protein sample are available (ESI Tables S10–S12†).

We included a horizontal line on the plots marking the maximum RFU seen in the CFG data. For 17% of the protein samples (213 of 1257 cases), at least one glycan is predicted to have a response more than one log(RFU) unit higher than that any of the recorded CFG glycans. In other words, the model predicts 10× more binding from one or more novel glycans, than from any glycan the model was trained against. Similarly, in 4% of the protein samples (46 out of 1257 cases), GlyNet predicted glycans with RFU more than 100 times higher than RFU or any recorded CFG glycan (see ESI Table S13† for a list of all responses). We analysed the ten glycans predicted to have the strongest binding for each output and observed that the distribution of strong binders was skewed towards a specific subset of the glycans. Of the 4160 glycans, only 750 glycans (18%) were present in any of the predicted top-10 binder lists (these glycans are listed in ESI Table S14†), while the remaining 3410 glycans never appear. Within the 750 glycans, the distribution was further skewed, with 50 glycans appearing in the top-10 of 46% of the samples (ESI Table S14†). The majority of these 50 glycans belong to only 4 or 5 distinct classes of glycans (ESI Fig. S7†): high-mannose structures (10/50), tri- and tetra-antennary *N*-glycan structures with *N*-terminal sialylation (16/50 including 6× α2–3 and 2× α2–6 sialylation), LacDiNac (4/50), LacNac (25/50), and 12 glycans that contain repeats of Gal(α1–3) (4/50), GalNAc(β1–4) (4/50) or 2-6-sialoLactose repeats-[Neu5Ac(α2–6)]Gal(β1–4)GlcNAc(β1–3) (4/50). Remarkably, the 2–6 sialolactose repeat glycans were predicted to be top-10 binders for 505 out of 1257 protein samples (45%). The privileged binding of only a few classes of glycans might be the result of natural binding preferences or it might represent bias in the training data skewing the GlyNet model or the encoding strategy favouring these types of glycans over the others. While experimental validation of these predictions over all 4160 glycans is infeasible, experiments using glycans from this group of 50 or from select members of the 4–5 classes may be a practical way to assess these predictions and gain assurance as to the general correctness of the predictions extrapolated by GlyNet.

We examined the binding preferences of the 4160 GlyTouCan glycans to the common lectins ConA, PNA, RCA, SNA I, and UEA I. We compared established binding motifs to the 20 glycan structures with the highest predicted RFU values. While agreement of the predicted strongly high-RFU glycans with known binding motifs does not prove that GlyNet is correct, it does suggest that the model is capturing patterns of glycan–protein behaviour seen previously by others. Although the 4160 glycans were different from any of the glycans on the CFG array (used for training GlyNet), the majority of the 20 glycans with the strongest predicted binding to these lectins contain the canonical binding motifs reported by Mahal and coworkers^36^ (ESI Fig. S8–S12a and d†). That the GlyNet model identifies structures with these canonical motifs as strong binders is obvious from their presence, often with multiple copies, in so many of the 20-strongest binding glycans (all 20 SNA I, RCA, and ConA; 17 of 20 PNA; 18 of 20 UEA I). For PNA, the three exceptions contain “near-misses” with Gal(β1–4)Gal or Gal(α1–4)GalNAc instead of Gal(β1–4)GalNAc, whereas the UEA I cases without the canonical UEA I binding motif, match the experimentally observed RFU values of a particular UEA I preparation (from EY Laboratories) to poly–GlcNAc oligomers (ESI Fig S14†). Examination of UEA I data reveals that the non-canonical binding preferences of UEA I occurs reproducibly in experiments that use UEA I protein from EY Laboratories but not protein from Vector Laboratories. This divergence is thus the result of the different preparations of UEA I protein from the two suppliers (ESI Fig S13 and S14†). The glycan binding preferences extrapolated by GlyNet, in turn also have two classes of predictions: one for UEA I from Vector Laboratories and the other for UEA I from EY Laboratories (the latter containing the non-canonical motifs). Although the molecular basis for this variation between these two sources is unknown, the target-agnostic GlyNet model needs no details of the molecular composition of the target, and it extrapolates divergent glycan-binging preferences for two sources (ESI Fig S14†).

### Comparison with other machine learning models

The original report of GlyNet in a BioRXiv preprint^44^ compared GlyNet with CCARL by Coff *et al.*^31^ and SweetTalk by Bojar *et al.*^32–35^ We expand this comparison by adding glyBERT,^46^ another model recently reported.

**Fig. 8:**
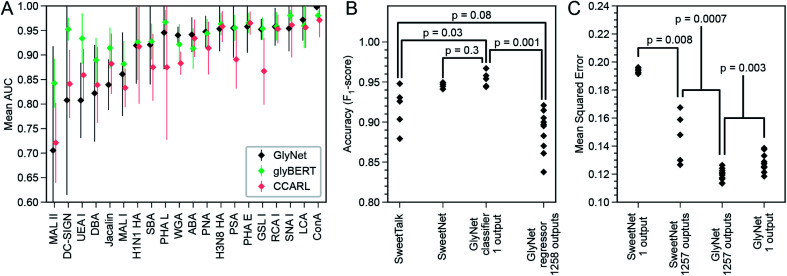
Comparison of GlyNet with CCARL, SweetTalk, SweetNet, and GlyBERT. (A) Performance of GlyNet, CCARL and glyBERT evaluated using Area Under the ROC Curve (AUC) on CV-folds for 20 proteins; while CCARL obtained a mean AUC value of 0.895, GlyNet-class obtained a better mean value of 0.912 as well as outperforming CCARL on 13 of the 20 examples; error bars are ±1 standard deviation. (B) Comparison with SweetTalk and SweetNet on the immunogenicity data. Individual points are *F*_1_-scores for the different hold-out folds. Reported *p*-values from comparing *F*_1_-score distributions by Mann–Whitney *U*-test (SweetTalk, SweetNet, and GlyNet 1-output *n* = 5; 1258 outputs, *n* = 10; ST *vs.* 1 output: *U* = 2, common language effect size *f* = 0.08; SN *vs.* 1 output: *U* = 7, *f* = 0.28; ST *vs.* 1258 outputs: *U* = 10, *f* = 0.2; 1 output *vs.* 1258 outputs: *U* = 0, *f* = 0). (C) Comparison with SweetNet on the CFG glycan array data using both single- and multi-output networks. Reported *p*-values are from comparing distributions of MSEs of cross-validation hold-out folds by M.-W. *U*-test (SweetNet *n* = 5; GlyNet *n* = 10; SN 1- *vs.* multi-output: *U* = 0, *f* = 0; GN 1- *vs.* multi-output: *U* = 12, *f* = 0.12; SN *vs.* GN multi-output: *U* = 0, *f* = 0). Note the GlyNet distributions are the same as in Fig. 4B.

The CCARL tool^31^ was trained on the same CFG glycan array datasets as GlyNet but with slightly different pre-processing. The authors binned the glycans by RFU values into low, medium, and high classes and then trained and evaluated the classifier on the simplified task of low-*vs.* high-category predictions, where the medium group was removed, *i.e.*, used neither in training nor in testing. To compare GlyNet to this tool, we created a classification version of GlyNet, GlyNet-Class, by replacing the activation functions on the output neurons with logistic functions that were then thresholded at their midpoint, ½, to divide the output between the low- and high-binding categories. (We used on the same test set as CCARL so the medium class was absent). We tested this modified GlyNet on the 20 proteins from CFG reported for CCARL, using the same 5-fold cross-validation sets as CCARL. GlyNet-Class yielded an AUC of 0.912, which was higher than CCARL's AUC of 0.895 (Fig. 8A and B). GlyNet-class, thus, not only can achieve a better performance after the simple conversion but also shows the flexibility of the GlyNet architecture. As GlyNet is a regression model, it can be converted to perform classifications but a classification model, like CCARL, cannot be simply converted into a regression model. Given that CCARL is reported to outperform GLYMMR,^56^ Glycan Miner,^57^ and MotifFinder,^58^ by transitivity GlyNet outperforms these tools too.

A possible explanation for GlyNet's superior performance may originate from the features it uses. The CCARL models used logistic regression on subgraph features after a carefully constructed set of steps selects which features to use. In contrast, GlyNet uses the full set of substructures (up to size 3) and the training of the neural network determines how to best use these features; an irrelevant feature will be effectively ignored once the learning process assigns it a small weight. This approach avoids the risk of handicapping the final regression stage by omitting a useful feature because the earlier processing failed to identify it as important.

SweetTalk was developed by Bojar and coworkers.^32–34,43^ Its classification of the immunogenicity of glycans is similar to GlyNet's prediction of RFU values, as a question of whether a glycan is immunogenic can be conceptually equated to whether that glycan interacts with immune signalling proteins. To compare GlyNet and SweetTalk, we assessed classification of SweetTalk's set of immunogenic glycans and an equal number (684) of randomly chosen human (non-immunogenic) glycans. Inspired by the improvements in performance we saw in the multi-task RFU outputs, we added an immunogenic prediction alongside our RFU outputs. We expanded GlyNet's set of features in the glycan “fingerprints” to accommodate additional monosaccharides and other *q*-grams not in the CFG set, then implemented a multi-output network version, that simultaneously output the 1257 RFU values and also an additional output that was then thresholded to predict whether or not the glycan is immunogenic. We found its performance was slightly different from SweetTalk's, with only weak significance (*p* = 0.08, Fig. 8C). We also implemented a single-output classifier, using a sigmoidal activation function for predicting “immunogenic”. The mean accuracy of this version of GlyNet's prediction of immunogenicity was (*F*_1_-score as used by Bojar and coworkers) of 0.954, a statistically significant (*p* = 0.03, Mann–Whitney *U*-test) increase over both SweetTalk (mean of 0.915) and the multi-output case (*μ* = 0.888, *p* = 0.001), illustrated in Fig. 8C. There is also an improvement over the newer SweetNet (*μ* = 0.946, *p* = 0.3), but the conservative Mann–Whitney *U*-test does not judge this to be significant. That the single-task network outperforms the immunogenic output of the multi-task case, suggests to us that the immunogenicity task is too divergent from the RFU tasks to be integrated effectively in our models with the available training data.

GlyNet and SweetTalk use a subtly different encoding for the network input: while both decompose glycans into subunits of three monosaccharides and the two linkages between them, there are differences. For example, GlyNet also includes smaller features, allowing it to process disaccharides or even monosaccharide inputs and more importantly, it maintains the branched character of glycans with branched structures – *i.e.*, we consider “V-structure” trisaccharides (Fig. 3E). To use the LSTMs of the SweetTalk system, the glycan structure is forced into a linear sequence with the end of one branch being immediately followed by the start of another. The feedforward network architecture of GlyNet offers flexibility in expanding the input features. The most important distinction is the regression (GlyNet) *versus* classification (SweetTalk) approaches used and a breadth of protein–glycan recognition learning tasks. It will be important in the future to assess the performance of a SweetTalk-like LSTM architecture with regression outputs on the CFG glycan array data or very similar datasets that capture a variety of protein–glycan learning tasks.

In another recent work,^35^ Bojar and coworkers followed-up with SweetNet, which is a graph convolutional neural network (GCNN) architecture with Top-K pooling, global mean and maximum pooling, and single- and multi-sample dropout layers that use logistic and leaky-ReLU activation functions. The authors used SweetNet to learn (from glycan bindings to 587 homologous viral coat proteins) a model that predicts the binding of a protein (learned as an amino acid sequence input to an LSTM model) to a glycan (learned using a GCNN). To compare this system to ours, we consider the SweetNet GCNN's performance on our dataset of 1257 samples across 599 glycans measured by CFG arrays. As many of these samples do not have an amino acid sequence and for better comparison with our task, we “turned off” the amino acid LSTM input (see ESI†) and evaluated only the GCNN glycan input. The hold-out MSE (from five-fold cross validation) of SweetNet, using 1257 single-output GCNN models, one per sample, was 0.194 ± 0.002 (Fig. 8D). The architecture is readily adapted to a variable number of outputs; we did so and produced a single multi-output GCNN model that took a glycan as input and produced 1257 outputs – like our multi-output GlyNet model. This system was more accurate (MSE = 0.146 ± 0.018; see Fig. 8D and ESI S15†) and significantly more efficient to train – ∼1200× faster than the 1257 single-output GCNN models. This shows that the benefits of a multi-output architecture that we saw for our architecture—improved MSE and a reduced training time—are even more pronounced for the SweetNet GCNN than for GlyNet, suggesting that the multi-output design is of general utility. While these tests show that the GlyNet design outperforms the SweetNet GCNN architecture (see Fig. 8C and ESI Fig. 16†), note that we tuned GlyNet-NN's hyperparameters and architecture for predictions on this CFG microarray data, whereas the SweetNet hyperparameters were optimized for a related task, and we used the SweetNet learning system “as is”. A full optimization of all the SweetNet system (including its learning algorithm, early stopping, annealing schedule, initialization, network architecture, *etc.*) may well find superior parameters, but this is beyond the scope of this paper. Finally, it is likely that the difference between the network architectures of the two systems means that there are some patterns in the data more readily learned by one system than by the other – which suggests that an ensemble or hybrid model, combining both systems, will work better than either.

A recent modification of SweetNet replaces its protein sequence input with ESM-1b,^59^ an amino acid sequence model developed using diverse proteins. Named LectinOracle,^45^ the revised system takes both an amino acid sequence and a glycan structure as inputs and outputs the predicted binding between the pair. In the task of predicting the predominant binding motif, the authors report that LectinOracle is superior to GlyNet. As the motif prediction is not the goal of GlyNet, the mismatch in performance warrants further investigation. As the glycan input layers in LectinOracle are reported to be the same as in SweetNet, we expect the performance on the RFU prediction task discussed here to be unchanged.

Reported in a recent preprint, glyBERT^46^ is another model of glycan–protein binding. Each glycan is decomposed into individual monosaccharides whose location in the glycan graph is represented by “subway” encoding and included as part of the network input to a BERT^60^ deep learning architecture. While a comparison of a small set of lectins (see Fig. 8A) shows that glyBERT and GlyNet are typically equal within the experimental noise, there are a small number of cases for which glyBERT is more consistent. Its authors' attribute glyBERT's performance to the attention mechanism provided by the BERT architecture.

## Summary and conclusions

GlyNet is a neural network architecture trained on glycans encoded with a straightforward method that respects their branched structures, to produce a model that can accurately predict the binding behaviours of glycans to proteins observed in glycan arrays. The multi-output architecture allows the training and optimization across thousands of protein samples at once and decreases the training time, which allows for a more extensive hyperparameter search. GlyNet outputs binding properties (*i.e.*, RFUs) directly whereas many previous approaches instead only predict a classification, or the sub-motifs considered responsible for binding.^18,19,22,23,25,27,28,30,31,57,61,62^ While it is possible to use general motif information to learn an accurate model for predicting binding, this still requires an additional learning process, which is non-trivial. GlyNet is a regression model (returning a continuous range of real-valued binding strength estimates, rather than binder/non-binder classification) and we believe that a shift towards regression, instead of just classification, is a critical improvement in protein–glycan interaction predictions as it provides more information about the interaction allowing a deeper analysis of the outputs. Moreover, if a classifier is desired, it is straightforward to threshold a continuous output. We believe that there is no reason to avoid building systems that predict continuous variables describing how strongly a glycan binds to specific proteins. This approach removes important decisions about choosing concentrations and binding thresholds from the machine learning pipeline and puts them into the hands of end users.

Our models have been developed using RFU measurements from CFG glycan arrays as proxies for true glycan–protein binding strengths. As such the techniques we use may be most appropriate for this dataset. However, we are confident that this approach is more generally applicable, and is useable not just for measurements from the many other styles of glycan arrays, but even other types of glycan–protein interaction measurements such as ITC when appropriate datasets exist.

We anticipate the value of the GlyNet model is two-fold. First, we hope that the techniques we describe will contribute to later, more accurate models. Second, GlyNet allows virtual screening of GBPs against large libraries of glycans, as we have demonstrated with the set of GlyTouCan glycans. The model estimates the behaviour of these glycans that were not included in the array, and we effectively have virtual arrays of over four thousand glycans which have been screened against all 1257 samples. The cost of extracting/synthesizing such a large number of glycans likely prevents such an array from ever being assembled in practice. The model provides reasonable speed, and competitive accuracy in its results. Further, the continuous numeric outputs can be compared with each other, allowing the glycans with the strongest projected binding to be identified.

The number of possible glycans is vast—even the number of small glycans containing only the most common monomers is orders of magnitude more than the number of glycans ever synthesized or isolated: a very restricted count of possible tetra- and penta-saccharides gives 10^6^ and 10^8^ glycans, but our training set of 599 glycans (with 385 composed of five or fewer monosaccharides) covers less than 0.0001% of this space. Still, our experiments show that this dataset allowed us to accurately estimate binding strength for most input glycan structures: for CFG-like glycans, predict the logarithmically transformed RFU values with a cross-validated MSE of 0.120. This is the first example of this type of (multi-output regression) for this RFU-prediction task. As future machine learning approaches are applied to create regression models in the same data, our MSE of 0.120 will serve as an important reference. Many of the 1257 samples are of the same protein at different concentrations, or of nominally the same protein from different sources. A future model may be able to group such samples together to learn a dose–response trend, but we have not directly explored this.

Glycan motifs are commonly used by the glycobiology community to roughly estimate the binding propensity without measuring the binding experimentally. In the absence of experimental validation, the analysis of the recurrent motifs is the most traditional approach for the evaluation of our predictions. In some cases (UEA-I, Fig. S13†) a post-prediction motif analysis yielded a previously overlooked divergence in the behaviour of lectin samples. Many of the false positives predicted by GlyNet are similar to “iconic recognition motifs; ” these mis-predictions may be because our training set did not include other examples of such “near misses”. Increasing the size of the datasets, specifically inclusion of nearly identical glycans with different protein interaction strengths would likely improve the performance. Moreover, we know that the CFG glycan array data contains some RFU values that are inconsistent with other data sources (false negatives).^63^ Poor prediction, thus, might be a result of suboptimal training data; however, this argument can only be supported with the inclusion of additional data. Both rationales point to the need for more data and potential strategic inclusions of interactions that focus on near neighbours of the binders.

One hazard of any data fitting approach to modeling is a risk of overfitting, where the model captures the specific data values it was trained from, rather than the underlying patterns in the data that allow it to generalize beyond the training set to the problem at hand. We guarded against this by testing each model on a subset (hold-out fold) of the data not used to develop the model, and requiring that any repeats of a glycan were assigned to the same fold, ensuring that the testing was never assessing the ability of the model to reproduce data for the glycans it was developed from, but instead that the outputs used to assess model quality were produced from a model trained against similar glycan structures.

In this work, we used a feed-forward neural network, although there are also more complicated systems such as graph neural networks. We did not use them in part because we wanted to quantify the accuracy of this simpler system to predict the protein–glycan binding for this class of data, RFUs from CFG glycan arrays. Importantly, its results are fairly strong. Another factor motivating our preference was the relatively small amount of training data. More complex variants of GlyNet (*i.e.*, architectures with multiple layers, or more nodes in hidden layers) may require more input data to train; indeed, our observation (see Fig. 4C and D) is that we saw some reduced performance in MSE in protein–glycan binding when layers and nodes were added even though these are strictly supersets of the case we did use.

The GlyNet model, like all published-to-date machine learned models built from glycan array data,^20,21,27,29–34,43,45,58,64^ is trained by data from specific preparations of the receptors (glycan-binding samples) and conditions in the array experiment. While RFU values vary with receptor preparation and array type^64,65^ the overall trends in such sets of RFU values are an important foundation of modern glycobiology^66^ from which much of the contemporary knowledge of protein–glycan interactions is inferred, of the form: “based on the experimental observation of how receptor *R* binds (or does not bind) to a set of glycans on the array(s), I predict that this receptor *R* should also bind to glycans G1 and should not bind to glycans G2 (where neither G1 nor G2 were present in the training arrays).” Our learned GlyNet model can autonomously perform the same inference while minimizing the requirement for human expert knowledge. The GlyNet model is learned from minimally processed experimental datasets, which could originate from multiple array types and receptor preparations; this model can extrapolate the semi-quantitative interactions of these receptors with any glycan, not just those tested on the experimental array. The current GlyNet system can deal with any glycan composed of the *q*-grams in the CFG dataset. We are currently exploring variants using atom-level *q*-grams, which will potentially allow a much broader inference of binding.

Our GlyNet model is “target-agnostic,” in that it does not need specific details of the composition of the possible receptor – n. b. some samples have uncertain composition (labels such as “Week 14 Conjugate-1 anti-Man4 Serum”). This contrasts with other machine learning models and molecular mechanics models (docking) that require knowing the exact composition of a receptor.^36,43,54^ We note that predictions for historical samples are challenging to validate without direct access to the exact materials used for the original data. Looking forward, combining serum profiling by glycan arrays and target agnostic prediction models might enable a better understanding of the interactions of glycans with constituents of complex serum samples. Specifically, it might allow extrapolating the interactions of sample components with biologically relevant structures—complex O– and N–linked glycans on cell surfaces and circulating glycoproteins and glycolipids—that might not be experimentally feasible to include on the glycan array.

It is likely that the next generation of glycan–protein binding models will outperform all contemporary models. One possibility for these may be hybrid architectures incorporating features of more than one of the existing models. In the SweetNet/LectinOracle case, just as the protein input layers were replaced, similar systems can be produced by just replacing the glycan input layers with more powerful systems, so experiments with hybrid systems using GlyNet or glyBERT^46^ style architectures for the glycan input are an obvious next step. Other architectures worth considering are hybrids of GlyNet's *q*-grams with the position-in-graph information of glyBERT, or with an attention mechanism, such as the BERT architecture.

More complex systems may require more input data to train; indeed, our empirical data suggest that the additional complexity of even a second or third hidden layer (see Fig. 4D) prevents results as good as the single layer case. Note that the good results of GNNs^67–69^ (on molecular structure graphs) were obtained on large training datasets, *e.g.* ∼250 000 molecules from the ZINC database;^70^ and that this dwarfs the 599 glycans in the CFG data we used. Our model represents protein–glycan binding across a diverse set of 1257 samples with a latent space of only 100 hidden neurons. That so many outputs can be constructed from linear combinations of this small number of hidden neurons is somewhat surprising and shows that many of the GBPs have great similarities in their binding patterns.

One feature of our system is its use of *q*-grams to encode glycan structures. While this has proven effective, there are three limitations of *q*-grams: (1) glycans that contain monomers that are not part of the training data are cannot be accurately represented; this problem is general to other approaches that use monomer and linkage information, and could be overcome by atom-level encodings. (2) Available training sets do not contain all possible *q*-grams. Compared to the lists of possible glycan structures mentioned in the introduction (see ESI Table 1†), the CFG dataset we use contains examples of 44 dimers and 103 trimers *versus* 840 and ∼57 thousand, respectively in the lists of possible glycans. (3) As implemented, the *q*-gram encoding represents only the connectivity between monomers (primary structure) but it does not contain any information about positions in space (geometry) between components, such as dihedral angles (secondary structure). This means glycan conformations are not part of the current encoding, however this might be done by introducing *q*-grams of different conformational states. It is possible that including information such as 3D-structure and dihedral angle preferences of glycans would improve the quality of the predictions. The caveat is the need for reliable access to such information for training. Comparison of the performance of models that account for dihedral angle information or omit it is one of the important next steps in development of useful machine learning models in glycobiology.

As of today, few datasets of protein–glycan interaction are organized in machine-readable, user-accessible formats; the CFG data used in this report is an exception, and a rich resource for training, albeit with limits in the size and number of CFG arrays and datasets. The next generation of models will need to integrate multiple data sources, as demonstrated by Klamer, Haab, and coworkers' CarboGrove,^64^ and even multiple measurement techniques (*e.g.* glass array^5,6^*vs.* bead-based array^71^*vs.* frontal affinity chromatography^72^). Comparing techniques shows cross-platform variability in the data. Today, this is often seen when high-throughput, medium-quality data is accompanied by high-quality thermodynamic and kinetic data acquired by low-throughput techniques, such as isothermal titration calorimetry or surface plasmon resonance. A next-generation ML model that can extrapolate the high-quality results across the moderate-quality set would be a valuable tool. It is worth noting that RFU values are usually compared within a single array experiment. Issues arise when comparing RFU values across multiple datasets, and such comparisons are frequently not meaningful. There are methods to counteract this, such as simple rank-based approaches (*c.f.*Fig. 3 and 5–7) as well as more sophisticated approaches developed for cross-platform (different glycan array technologies) comparisons.^63^ We foresee three problems *en route* to such a tool and other next-generation ML models: (i) the data science problem of identification and organization of the needed data, currently residing in assorted publications. Advanced techniques for mining and extraction of data (from PDFs) are needed to avoid labour-intensive and error-prone manual extraction of this data. (ii) The biochemical problem of describing of glycan presentation—for example valency, spacing, mobility, and solution *vs.* surface immobilized—and encoding this so that machine learning algorithms can use it. (iii) The machine learning problem of selecting effective learners that will yield useful regression models starting from noisy inputs of diverse quality and variability. Several other important directions are simultaneous representation of proteins and glycans (first examples recently shown by Bojar *et al.*^35,45^) as well as all-atom representations of glycans with the goal of including binding affinities of glyco-mimetic compounds and non-glycan structures into training datasets. Advances in building predictive models of protein–glycan interactions requires open datasets with transparent sharing of algorithms to follow the successful path of AlphaFold2's protein structure prediction built on PDB, CATH, psiBlast nr, and UniClust—all public datasets.^73^

## Data availability

Copies of the code used are available at https://github.com/derdalab/GlyNet.

## Author contributions

R. D. and S. S. conceived the original concepts and methodology. E. J. C., S. S., and N. Y. developed GlyNet, curated data, performed analysis and visualized results. R. G. and R. D. supervised the work. All authors wrote, reviewed, and revised the manuscript.

## Conflicts of interest

There are no conflicts to declare.

## Supplementary Material

SC-013-D1SC05681F-s001

SC-013-D1SC05681F-s002

SC-013-D1SC05681F-s003

SC-013-D1SC05681F-s004

SC-013-D1SC05681F-s005

SC-013-D1SC05681F-s006

SC-013-D1SC05681F-s007

SC-013-D1SC05681F-s008

SC-013-D1SC05681F-s009

SC-013-D1SC05681F-s010

SC-013-D1SC05681F-s011

SC-013-D1SC05681F-s012

SC-013-D1SC05681F-s013

SC-013-D1SC05681F-s014

SC-013-D1SC05681F-s015

SC-013-D1SC05681F-s016

SC-013-D1SC05681F-s017

SC-013-D1SC05681F-s018
