# Supplementary material for: GlyNet: a multi-task neural network for predicting protein–glycan interactions
Source: Chem Sci. 2022 May 16;13(22):6669–86. doi: 10.1039/d1sc05681f (PMC9172296; doi:10.1039/d1sc05681f)
Supplement: SC-013-D1SC05681F-s001 [file SC-013-D1SC05681F-s001.pdf]

Electronic Supplementary Material (ESI) for Chemical Science.
This journal is © The Royal Society of Chemistry 2022

Supporting Information for

GlyNet: A Multi-Task Neural Network for Predicting Protein-Glycan Interactions

Eric J. Carpenter,[1] Shaurya Seth,[1] Noel Yue,[1] Russell Greiner,[2,3] Ratmir Derda[1]*
1. Department of Chemistry, University of Alberta, Edmonton, Alberta, Canada
2. Department of Computing Science, University of Alberta, Edmonton, Alberta, Canada
3. Alberta Machine Intelligence Institute (AMII), Edmonton, Alberta, Canada

* corresponding author: ratmir@ualberta.ca

Table of Contents

1. Methods 2

1.1 Selection of CFG Glycan Array Protein Binding Data 2
1.2 Preprocessing 3
1.3 Glycan Representation for Input to the Neural Networks 3
1.4 Neural Network Architecture. 4
1.5 Trained model quality evaluated by 10-fold CV 4
1.6 Parameter Optimization 4
1.7 Comparison with Other Works 4
1.8 Statistics 5
1.9 Models on Expanded Dataset 6
1.10 Generation of lists of possible small glycans 6

2 Electronic Supplementary Information – Tables 7

2.1 ESI Table S1 – List of Possible Small Glycans 7
2.2 ESI Table S2 – List of Glycans Used 7
2.3 ESI Table S3 – CFG Array Information 7
2.4 ESI Table S4 – Mean RFUs (reference from CFG) 7
2.5 ESI Table S5 – Glycan Feature Counts 7
2.6 ESI Table S6 – Linkers 8
2.7 ESI Table S7 – Reproduction of Hold-out Data 8
2.8 ESI Table S8 – Protein Validation – Animation Time Index 8
2.9 ESI Table S9 – Glycan Validation – Animation Time Index 8
2.10 ESI Table S10 – Complete Training of GlyNet 8
2.11 ESI Table S11 – Single Output Network Predictions 8
2.12 ESI Table S12 – Protein Predictions – Animation Time Index 8
2.13 ESI Table S13 – Novel Glycan Extrapolation 8
2.14 ESI Table S14 – Number of Top-10 Counts by Glycan 8

2.15 ESI Table S15 – Single-output vs. Multi-output Network Predictions 9
3 Electronic Supplementary Information – Figures 10

Fig. S1 – High-resolution RFU Heatmap with Dendrograms 10
Fig. S2 – Isomeric glycans with identical glycan feature counts 11
Fig. S3 – MSE vs. R^2 12
Fig. S4 – Correlation between MSE, R^2 and properties of the data 14
Fig. S5 – Examples of low-MSE-low-R^2 data 15
Fig. S6 – Correlation between MSE, R^2 for multi-output vs single output GlyNet 16
Fig. S7 – The Top 50 Glycans 17
Fig. S8 – Glycan Binding to SNA 20
Fig. S9 – Glycan Binding to ConA 21
Fig. S10 – Glycan Binding to PNA 22
Fig. S11 – Glycan Binding to RCA 23
Fig. S12 – Glycan Binding to UEA I 24
Fig. S13 – Patterns of Glycan Binding to UEA I in the CFG Data 26
Fig. S14 –Glycan Binding to UEA I 28
Fig. S15 – Analysis of Predictions from the SweetNet Multi-output Models 30
3.1 Fig. S16 – Comparison of MSE Distribution between the SweetNet and GlyNet Models31
4 References 31

1. Methods

1.1 *Selection of CFG Glycan Array Protein Binding Data*

A list of glycan array datasets was obtained from a search of the CFG website. Available CFG data was downloaded for all v5.0, 5.1, 5.2 Mammalian arrays. After minor corrections of the links, in particular duplicate link removal, data was downloaded from URLs of the form: http://www.functionalglycomics.org:80/glycomics/HFileServlet?operation=downloadRawFile&fileType=DAT&sideMenu=no&objId=1006594

where the final seven digits (the objId) varies between samples. See Supplementary Table S2 for a list of the samples and their objId numbers.

The downloaded files are MS Excel Workbooks, and we extracted the source data from the ImaGene file formatted table. Datasets without this table were excluded from this work. The RFU for each replicate was calculated as: Mean.Signal – Mean.Background (both columns in the ImaGene table) which matches CFG's processing described in the MS Excel files. Mean and standard deviation of these RFU values were calculated across the six replicates. This produces a wider dispersion than CFG's numbers. Additional alignment, blank spots, and reference signals IgG, etc., were not used.

We omitted some data. Of the 611 glycans on the arrays:

i) we omitted 2 glycans not present in all three versions of the arrays,

Neu5Ac(α2-6)Gal(β1-4)GlcNAc(β1-2)Man(α1-6)[Neu5Ac(α2-6)Gal(β1-4)GlcNAc(β1-2)Man(α1-3)]Man(β1-4)GlcNAc(β1-4)GlcNAc(β-Sp13

GlcNAc(β1-2)Man(α1-6)[Neu5Ac(α2-6)Gal(β1-4)GlcNAc(β1-2)Man(α1-3)]Man(β1-4)GlcNAc(β1-4)GlcNAc(β-Sp12

ii) we omitted 1 glycan structure reported with a broken (unbalanced and therefore unparsable) branching pattern,

Gal(β1-4)GlcNAc(β1-6)[Gal(β1-4)GlcNAc(β1-2)]Man(α1-6)[GlcNAc(β1-4)]Gal(β1-4)GlcNAc(β1-4)[Gal(β1-4)GlcNAc(β1-2)]Man(α1-3)]Man(β1-4)GlcNAc(β1-4)[Fuc(α1-6)]GlcNAc(-Sp21

iii) we omitted 1 glycan structure reported to contain …Man(α1-3)[... Man(α1-3)]Man… (two substructures both bound to carbon-3),

Fuc(α1-4)[Fuc(α1-2)Gal(β1-3)]GlcNAc(β1-2)Man(α1-3)[Fuc(α1-4)[Fuc(α1-2)Gal(β1-3)]GlcNAc(β1-2)Man(α1-3)]Man(β1-4)GlcNAc(β1-4)GlcNAc(β-Sp19

iv) we omitted 8 glycans that contained one of five rare sugars (four or fewer instances within the CFG glycan set).

Rha(α-Sp8,   GlcN(Gc)(β-Sp8,   G-ol(-Sp8,   MurNAc(β1-4)GlcNAc(β-Sp10

Neu5,9Ac2(α-Sp8,   Neu5,9Ac2(α2-6)Gal(β1-4)GlcNAc(β-Sp8

Neu5,9Ac2(α2-3)Gal(β1-4)GlcNAc(β-Sp0,   Neu5,9Ac2(α2-3)Gal(β1-3)GlcNAc(β-Sp0

We use the remaining 599 glycans and a full list of the glycans is in Supplementary Table S3. There are some minor differences in glycan name/structures found in different files, we used the latest version of the name/structure, which often corrected obvious errors in earlier versions. Our analysis ignores the linkage/spacer attaching each glycan to the glass array and the stereochemistry of the anomeric position (alpha or beta) to which the linker is attached. We note that the removal of the stereochemistry of the anomeric carbon impairs the model as it cannot distinguish between cases such as Man(a1- and Man(b1- which have distinct binding properties. We also note that the information about the stereochemistry of the anomeric position is not available for 105 out of 599 glycans (see Supplementary Table S3).

Removal of the linker/spacer information creates some duplicates. Specifically, in a list of 599 glycans with linkers there are 520 unique glycan structures. After removal of linker, we treat glycans as distinct entities during training although our network cannot predict different results for them. For example, (3S)Gal(β1-3)GlcNAc(β-Sp0 and (3S)Gal(β1-3)GlcNAc(β-Sp8 are treated as identical and they are encoded using the same features (Supplementary Table S5). Further details of the duplicates are available in Supplementary Table S3.

1.2 *Preprocessing*

Initial preprocessing of the data was copied from Coff et al.[1]; a constant was added to each array's data so that the mean RFU minimum is one before transforming with $\log_{10}$. Then low-end noise was filtered out with a minimum clamp at the 1/3rd rank position.

1.3 *Glycan Representation for Input to the Neural Networks*

For the CFG glycan set there are 272 features, for a list see the columns of the fingerprint file (Supplementary Table S5). Features "S3", "S4", "S6", and "P6" are SO_4/PO_4 attached to carbons 3, 4, 6, and 6

respectively. Feature names beginning with '[' are monosaccharides located in terminal positions, these are also counted within the regular monosaccharide counts. As noted in Section 1.1 above, not included in the features and therefore not available to the model are details of the spacer attaching the glycan to the substrate and details of its attachment.

1.4 *Neural Network Architecture.*

- fully-connected feedforward neural network
- 0-3 hidden layers, all hidden layers of a common size
- ReLU activation functions, except for linear outputs at the final stage; all with biases
- feature count vector (fingerprint) to describe a glycan as network input
- list of RFU values, one for each of the protein samples as output
- implemented using PyTorch[2]
- ADAM[3] optimiser with weight decay and early stopping

1.5 *Trained model quality evaluated by 10-fold CV*

Folds were created by randomly dividing (unique) fingerprints (feature vectors). Equal numbers (±1) of unique fingerprints are assigned to each fold. This means that indistinguishable glycans (i.e. those with duplicate feature vectors) are assigned to the same fold. In particular, multiple instances of the same glycan distinguished by CFG with different spacers are grouped together. This prevents evaluating in the hold-out fold (i.e. the post-training test-fold) a glycan also in another fold and thus used for training. The exact number of glycans in each group varies. MSE was used to assess quality of training and CV.

1.6 *Parameter Optimization*

The ensure comparability when testing the effect of the number of outputs on the model, the MSEs are computed over the same set of 1200 protein samples for all output sizes. A random permutation of the protein samples was produced and to evaluate the 10-output case a first model using the first ten outputs was produced, a second model from the second group of ten, until 120 models had been created. The same permutation was also used for the other sizes. The reduction to 1200 outputs is because 1200 is highly composite, had all 1257 been used only comparable 1-, 3-, 417-, and 1257-output cases could be produced.

Using MSE and the in-fold results we tested zero through three hidden layers with 25 to 1100 neurons in each of them. By the MSE we found that a single hidden layer of 100 neurons produced an optimal MSE CV score (Fig. 4b). Similarly, we varied the ADAM weight decay parameter, while keeping 100 neurons per hidden layer and found the MSE performance was impaired as the weight-decay increased much beyond 10^{-4} (Fig. 4c). Accordingly, we fixed this parameter at 10^{-4} for subsequent work.

1.7 *Comparison with Other Works*

For binary classification tasks, the comparisons with CCARL and the SweetTalk immunogenicity single output, the final output layer of the neural networks are changed to logistic activation functions, and the output is then thresholded at ½ to produce the binary classification.

SweetTalk immunogenicity dataset: The 684 glycans in the immunogenic_glycans_clean.csv and 684 randomly chosen human glycans from glycol_targets_species_seq.csv dataset (presumed non-immunogenic). This dataset is the same as used by the SweetTalk developers, we retain some duplicate glycans present in both groups.

This data is added as an additional "glycan array" to the CFG data by using "artificial" RFU values of 4 and 1 (after log transformation) for the immunogenic and non-immunogenic glycans respectively. Processing this also requires expanding the list of features to accommodate the additional mono-, di- and tri-saccharides not found in the 599 CFG glycan set. To convert the predicted RFU values (hold-out fold) of for this dataset to the binary classes, they were thresholding at the mean (2.5) of the two RFU input values. Changing the threshold setting from this half-way point reduced the accuracy of the results.

To run the CFG data used in this paper on the SweetNet viral receptor prediction architecture[4], we needed to supress the protein sequence input. To minimize the changes needed to the code provided by Bojar and coworkers, we provided a single dummy sequence, "X" (i.e. a single ambiguous amino acid), for all cases. With no pattern between this constant input and the RFU outputs, no learning can occur. The other inputs were then the glycan graphs (using Bojar's notation convention) and the RFU values. Depending on whether we were training for the single- or multi-task case each glycan corresponds to either one RFU value (for the appropriate protein) or a list of 1257 values. Other changes that were made were a modification to enable using graphs with no edges (i.e. monosaccharides) and the removal of the prot_env variable. We also modified the code to learn on all five cross-validation folds and to output the results for all glycan-protein sample pairs.

1.8 *Statistics*

The RFU values obtained from CFG each have measurements are 6 replicate spots on the glycan array. We take the difference between the reported Signal Mean and Signal Background as the RFU for each spot, before calculating the mean RFU, μ, over all 6 replicates. A population standard deviation, σ, is calculated from the same six values. To estimate a 95% confidence interval the limits $\mu \pm 1.96\ \sigma$ are used, the symmetric 95% CI of a Gaussian having the mean and standard deviation of the replicates.

In Fig. 4 each marked data point is calculated on a different cross-validation fold. The mean and population standard deviation were calculated, and the range of the plotted error bars is the 95% confidence interval calculated with $\mu \pm 1.96\ \sigma$, as for the RFU values. Error bars are from a single set of predictions across the 10-fold cross-validation, with a total of N = 10 points.

For comparing sets of values (see Fig. 8), Mann-Whitney U-tests are used, and two-tailed *p*-values are reported. Common language effect sizes reported are calculated by $f = U / (n_1 n_2)$ where n_1 and n_2 are the sizes of the two sets being compared.

<u>Predictions of the top 10 or top 20 strongest binders</u>

Glycans from the set of 599 glycans or proteins from the set of 1257 protein samples were evaluated against a classic urn problem. For example, in Figure 5B-D, the model predicts top 20 strongest binding protein samples (from 1257 total) and it can predict a mean of 10.9 (95% CI: 6–15) of them correctly. How much better is this model than a random guess?

Given an urn with 1257 balls, 20 red and the rest white. The random model then draws 20 balls (without replacement). What is the chance that 0 of them are red? 1 of them is red? 2 are red? ... 20 are red? The answers are a hypergeometric distribution:

$$Pr(X = k) = K! (N - K)! n! (N-n!) / (k! (K-k)! (n-k)! (N-K-n+k)! N!)$$

Where N = 1257 balls total, K = 20 red balls, n = 20 draws, and k is the number of red balls drawn. Evaluating the performance of this random model at various k:
0 from the top-20: 72%
1 from the top-20: 24%

2 from the top-20: 3.5%

6 from the top-20: $2x10^{-5}$ % (lower edge of 95% confidence interval)
10 from the top-20: 1×10^{-12}% (average rounded down)
11 from the top-20: 9×10^{-15}% (average rounded up)
15 from the top-20: 1×10^{-24}% (upper edge of 95% confidence interval)

1.9 *Models on Expanded Dataset*
While most of our work has focussed on the dataset obtained from CFG's version 5 glycan arrays, we have also experimented with a larger dataset which included data for additional samples run on Mammalian Printed Array versions 2, 2.1, 3, 3.1, 3.2, 4.0, 4.1, and 4.2. As for the version 5 data. files (MS Excel formatted) containing the primary screening data were downloaded from CFG's website, and replicate spot data was then extracted from embedded ImaGene tables. Where the arrays had both 10 µM and 100 µM concentration parts, we used only data from the 100 µM spots.

The RFU data for each sample was pre-processed as described for the main dataset with the minimum value noise-cutoff set to affect one third of the glycans for each sample. Glycan names were matched to those in the version 5 arrays.

To make the data processing as similar as possible with the original version 5 array dataset, the resulting log-scaled RFUs were copied into the same list of 599 glycans as we used for the rest of this work. As not every glycan in the version 5 arrays has a corresponding glycan in the older ones, there are missing values, which have been filled-in with a "not available" marker. In practice, we found the value -1 to be useful for this, as it can be distinguished from the real log-scaled RFU values (all positive) by checking the sign.

This results in a table (samples × glycans) of known log-RFUs, but which has some holes, locations where we do not have a value from experimental observations. We can use table in our existing learning architecture, but it is necessary to prevent the backpropagation step from trying to drive the prediction outputs at these missing values. In order to do this, we modified the loss (squared error) function by adding a mask:

$$\text{Loss} = \text{mask} \times (\text{predicted} - \text{actual})^2,$$

where the mask is zero (no actual RFU value known) or one (actual data known). This forces the loss to zero when no measured value is available.

1.10 *Generation of lists of possible small glycans*
The lists of possible small glycans (ESI Table S1) were generated with a short program. It works by taking base structures and creating a list of all the positions at which another monosacharride may be added. Then at each of these positions the 10 monosacharrides are added in both alpha and beta conformations. These new, larger structures are then used as the base structures for another round of enlargement.

As a concrete example consider GalNAc(b1-3)Glc(b1-. There are six locations at which another glycosidic bond may be formed are: GalNAc oxygens 3, 4, and 6 and Glc oxygens 2, 4, and 6.

Because they are already in glycosidic bonds are both position 1's and Glc position 3 are not available. Similarly, GalNAc oxygen 2 is not available because of the NAc modification, and both oxygen-5s are unavailable as they are in the rings.

At each of the six sites available for glycosidic bonds, we can add each of the ten monosaccharides, GlcNAc, GlcA, Gal, …, etc. as either alpha or beta to produce 6 x 10 x 2 = 120 trisaccharides from this disaccharide.

The enlargement of the structures is restricted to positions 2, 3, 4, and 6 on the hexoses Gal, Glc, and Man. From GalNAc and GlcNAc only additions at 3, 4, or 6 are considered. GlcA allows extension at 2, 3, and 4, while Fuc, Kdn, Neu5Ac, and Neu5Gc have no sites for the extension of the structures.

In practice, the program has a few refinements to prevent producing duplicate structures and produces the output in a depth-first order.

2 Electronic Supplementary Information – Tables

2.1 *ESI Table S1 – List of Possible Small Glycans*

This table lists the possible mono-, di-, tri-, and tetra-saccharides we count in the introduction generated as described in Section 1.10.

2.2 *ESI Table S2 – List of Glycans Used*

This table contains details of the glycan identifiers used in the different data sources, this work, and the three versions of the CFG glycan arrays as well as the "Gene ID" used in the ImaGene style raw data tables. This is not actually a gene identifier, but the standard label used by the software which expects to analyze a DNA microarray. Entries without a number in the GlyNet column were not used in this work. Groups of glycans which are identically encoded in our methodology are assigned the same Unique Encoding number; this field is left blank for uniquely encoded glycans. Glycans for which anomer attached to the spacer is unspecified are marked with a question mark in the "Unknown Anomer" column. See Supplementary Methods 1.1 for rationals.

2.3 *ESI Table S3 – CFG Array Information*

This table lists the 1257 glycan array datasets used in this work, along with various metadata extracted from the CFG website including descriptions of the protein sample used.

2.4 *ESI Table S4 – Mean RFUs (reference from CFG)*

Contains the mean RFU values after all the preprocessing steps. The table is 599 glycans x 1257 protein samples. These values are the ground truth used in learning, and used for the references for MSE calculations, and references in Figs. 4-7 and associated animations.

2.5 *ESI Table S5 – Glycan Feature Counts*

This table contains are list of the glycans and the counts of the features used to describe them to the neural networks. Apart from the header, each line of the file is a different glycan and its "fingerprint".

2.6 *ESI Table S6 – Linkers*

This table contains the list of unique linkers and their chemical structure. The chemical structures of the linkers are according to the information found at the CFG website (http://www.functionalglycomics.org/static/consortium/resources/resourcecoreh8.shtml).

2.7 *ESI Table S7 – Reproduction of Hold-out Data*

This table lists the GlyNet outputs (predictions) used in part to create Figs. 5 and 6 and produce the associated animated plots. This table reports output values (corresponding to hold-out folds) that compare to available CFG data (in ESI Table S5).

2.8 *ESI Table S8 – Protein Validation – Animation Time Index*

This table contains times and provides links at which different protein samples and occur within the animated plot analysing the CFG-validation outputs one protein per frame.

2.9 *ESI Table S9 – Glycan Validation – Animation Time Index*

This table contains times and provides links at which different glycans occur within the animated plot analysing the CFG-validation outputs with one glycan per frame.

2.10 *ESI Table S10 – Complete Training of GlyNet*

This table lists GlyNet outputs used to create Fig. 7 and produce the associated animated plots. Here the network has been trained on all the available data and is reproducing (after learning) the CFG RFU values.

2.11 *ESI Table S11 – Single Output Network Predictions*

Table with GlyNet outputs from the single-output network variants. These are used in Fig. 4 and compared with ESI Table S7 in ESI Fig. S6.

2.12 *ESI Table S12 – Protein Predictions – Animation Time Index*

This table lists times and provides links at which different glycans occur within the animated plots analysing the extrapolated predictions for the novel glycans.

2.13 *ESI Table S13 – Novel Glycan Extrapolation*

This table contains GlyNet outputs used to create Fig. 7 and produce the associated animated plots. Using the same learned weights, as in ESI Table S8, the network is extrapolating RFU values over the set of novel glycans.

2.14 *ESI Table S14 – Number of Top-10 Counts by Glycan*

This table contains statistics about how often different glycans appear as one of the ten strongest binding glycans for each protein sample.

2.15 *ESI Table S15 – Single-output vs. Multi-output Network Predictions*

Table of MSE and R^2 values evaluation of the predictions across glycans for each protein sample from both a single-output network and a shared multi-output network.

3 Electronic Supplementary Information – Figures

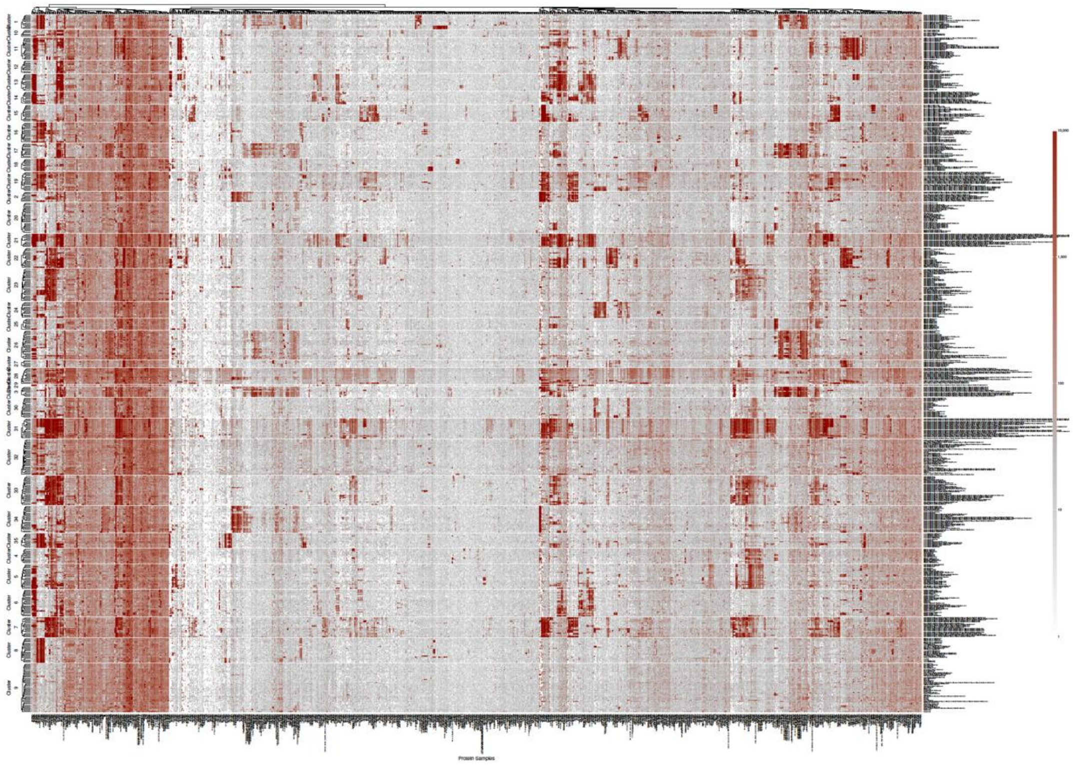

Top right corner (zoomed in)

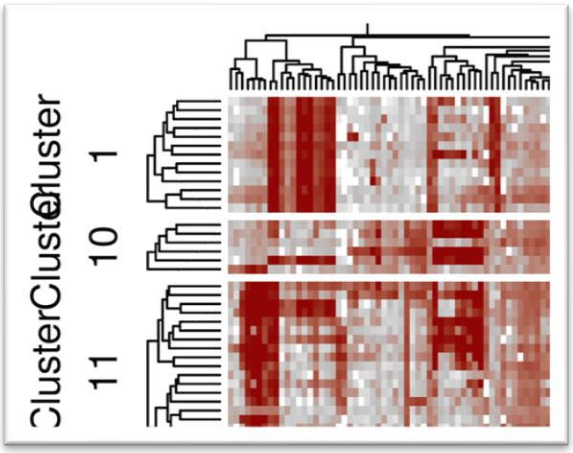

Bottom left corner (zoomed in)

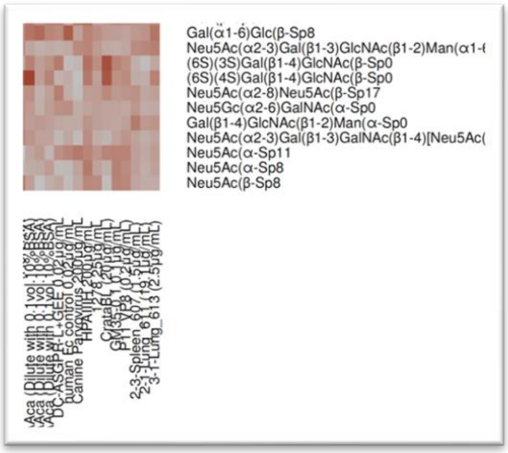

Fig. S1 – High-resolution RFU Heatmap with Dendrograms

Snapshots of the high-resolution version of the heatmap in panel Fig. 3a, this includes dendrograms showing clustering of the glycans and the glycan arrays (protein samples) as well as including textual labels for these items. Space limitations prevent these features from being included in Fig. 3a. The full figure is available in the supporting files folder as “Fig. S1 - RFU Data Heatmap with Dendrograms.pdf”

Isomer 1 **Isomer 2**

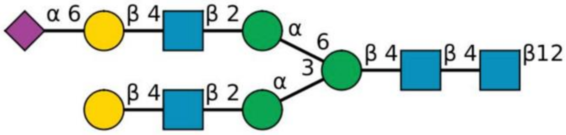

GlyTouCan: G75850OP
IUPAC: Neu5Ac(a2-6)Gal(b1-4)GlcNAc(b1-2)Man(a1-6)[Gal(b1-4)GlcNAc(b1-2)Man(a1-3)]Man(b1-4)GlcNAc(b1-4)Glc-NAc(b-Sp12

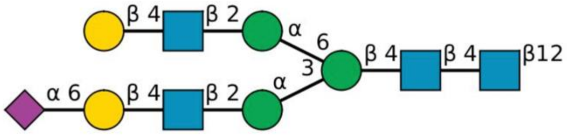

GlyTouCan: G91365ZQ
IUPAC: Gal(b1-4)GlcNAc(b1-2)Man(a1-6)[Neu5Ac(a2-6)Gal(b1-4)GlcNAc(b1-2)Man(a1-3)]Man(b1-4)GlcNAc(b1-4)Glc-NAc(b-Sp12

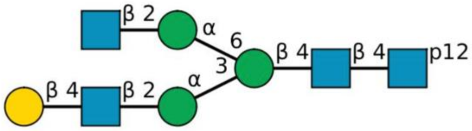

GlyTouCan: G99129GB
IUPAC: GlcNAc(b1-2)Man(a1-6)[Gal(b1-4)GlcNAc(b1-2)Man(a1-3)]Man(b1-4)Glc-NAc(b1-4)GlcNAc(-Sp12

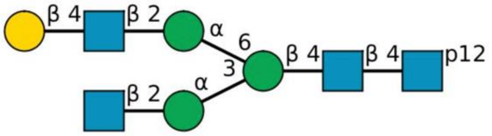

GlyTouCan: G86102WJ
IUPAC: Gal(b1-4)GlcNAc(b1-2)Man(a1-6)[GlcNAc(b1-2)Man(a1-3)]Man(b1-4)Glc-NAc(b1-4)GlcNAc(-Sp12

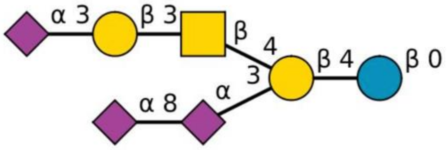

GlyTouCan: G40183QN
IUPAC: Neu5Ac(a2-3)Gal(b1-3)GalNAc(b1-4)[Neu5Ac(a2-8)Neu5Ac(a2-3)]Gal(b1-4)Glc(b-Sp0

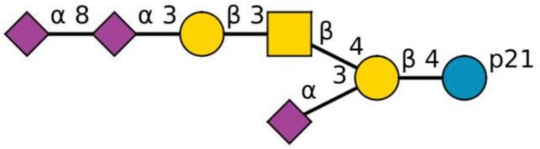

GlyTouCan: G97898ZO
IUPAC: Neu5Ac(a2-8)Neu5Ac(a2-3)Gal(b1-3)GalNAc(b1-4)[Neu5Ac(a2-3)]Gal(b1-4)Glc(-Sp21

Fig. S2 – Isomeric glycans with identical glycan feature counts

In the set of 599 glycans, we found three pairs of glycans (6 total) that are encoded by the same set of glycan feature counts. The Figure summarizes the structures, GlyTouCan codes and IUPAC names for these three pairs. Details of the encoding is available in Supplementary Table S5 - Glycan Feature Counts.xlsx

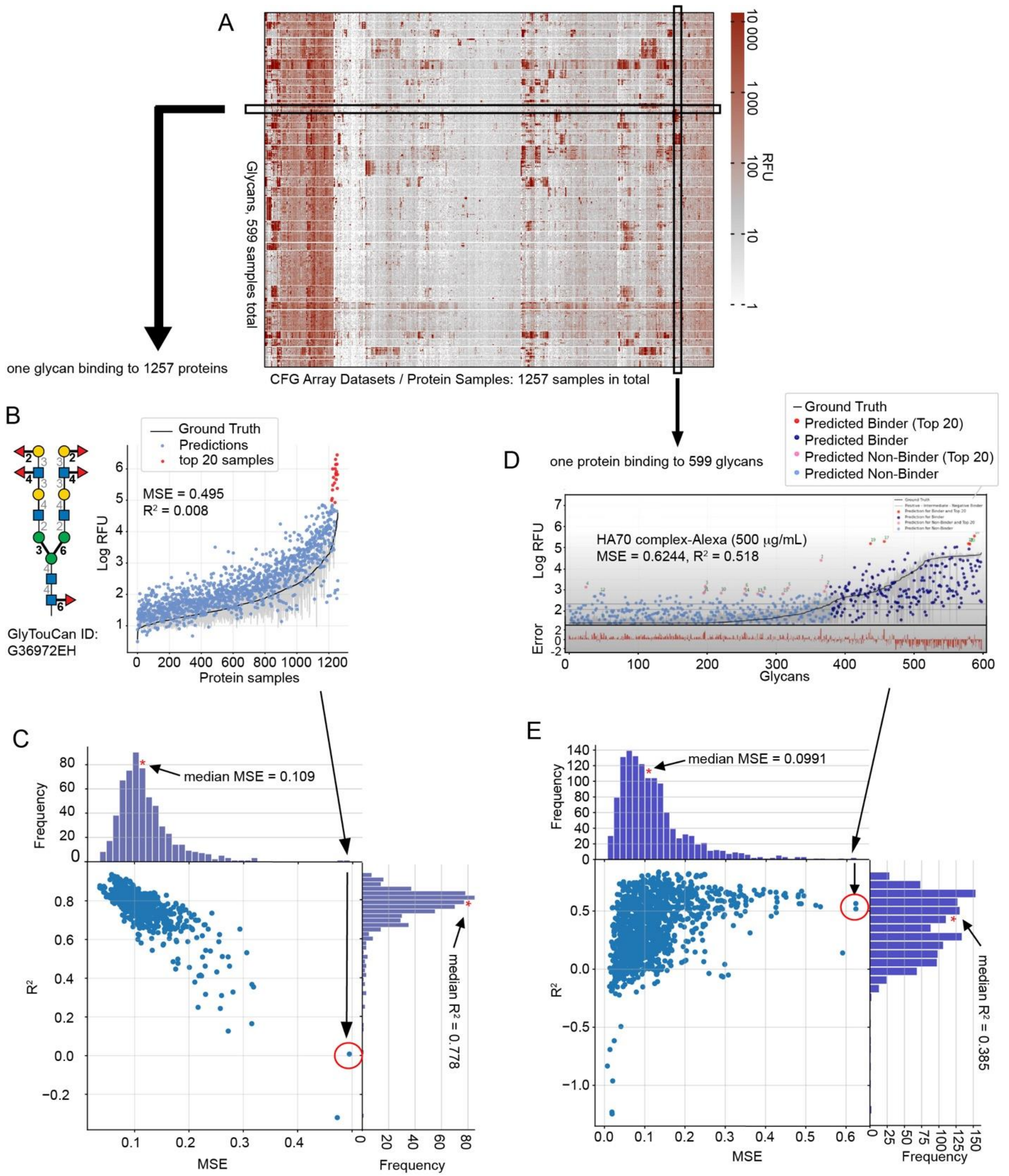

Fig. S3 – MSE vs. R^2

Comparison if the two measures of quality across the glycans and the protein samples. panel (A) is copied from the Main Text Figure 3A; the vertical and horizontal slice of the heatmap represent two ways of looking at the data: (B-C) a plot describing binding of one glycan to 1257 protein samples; there are 599 plots total, all summarized as a scatter of 599 dots in MSE vs. R^2 plot in panel (C) and available as 599-frame-long video at https://youtu.be/biWNApZHMP8

(D-E) a plot describing binding of one protein sample to 599 different glycans (1257 plots total). there are 1257 plots total, all summarized as a scatter of 599 dots in MSE vs. R^2 plot in panned C) and available as 1257-frame-long video at https://youtu.be/oHaFF4A22D8

(B) is an example of the worst prediction of Glycan G36972EH binding to 1257 with MSE=0.495 copied from Figure 5D and the location of this prediction on the MSE vs. R^2 plot (C) is indicated by an arrow. (D) is an example of the worst prediction for HA70 protein binding to 599 glycans and the location of this prediction on the MSE vs. R^2 plot (D) is indicated by an arrow.

Although median MSE values are similar across (B-C) and (D-E) the median R^2 values differ substantially. Furthermore, in (D-E) the values MSE and R^2 are not correlated and there are numerous instances of models with low MSE (low error) but also low R^2 (“poor fit”) and vice versa: high MSE (high error) but also high R^2 (“good fit”). Figures S3-S5 provide further details.

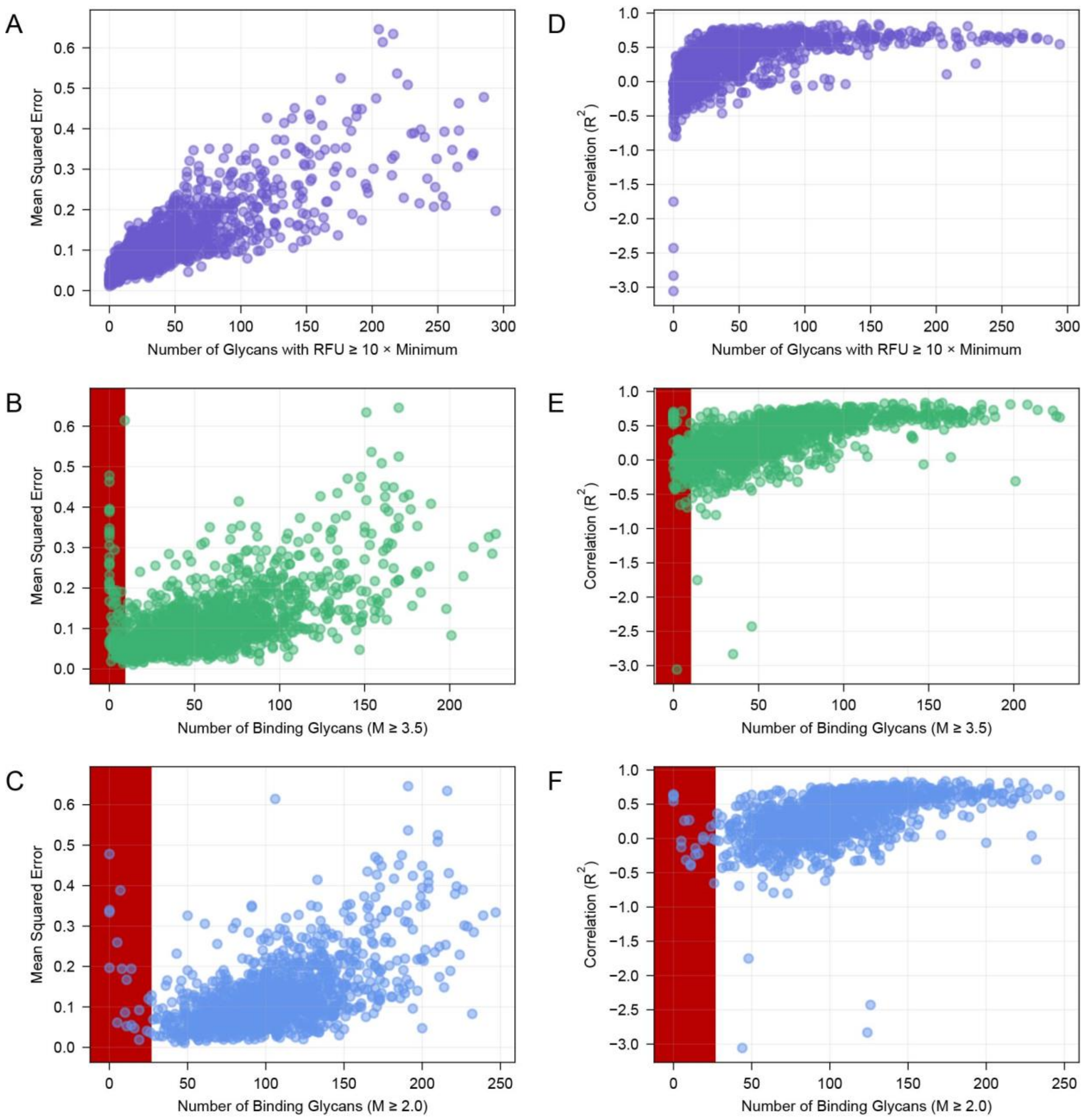

Fig. S4 – Correlation between MSE, R^2 and properties of the data

(A) MSE correlates with the number of binding glycans that exhibit a response 10x above the background "N_{RFU10}". The error of the fit is low when samples has low N_{RFU10} whereas increase in the N_{RFU10} in general increases the error of the fit. (B-C) There is a weaker correlation between MSE and the number of binding glycans N_{bind}; N_{bind} was defined using criteria of Coff et al.[1]. (D) in contrast to (A), the R^2 of the fit improves with increase in N_{RFU10}, whereas low R^2 is attributed exclusively to samples with no glycans that exhibit strong response (see Figure S3). (E-F) Samples with large number of binding glycans exhibit the best R^2 values whereas majority of the data with poor R^2 values can be attributed to samples N_{bind} < 50. Further details are Table S9 - Timestamp Indices for Animations.xlsx

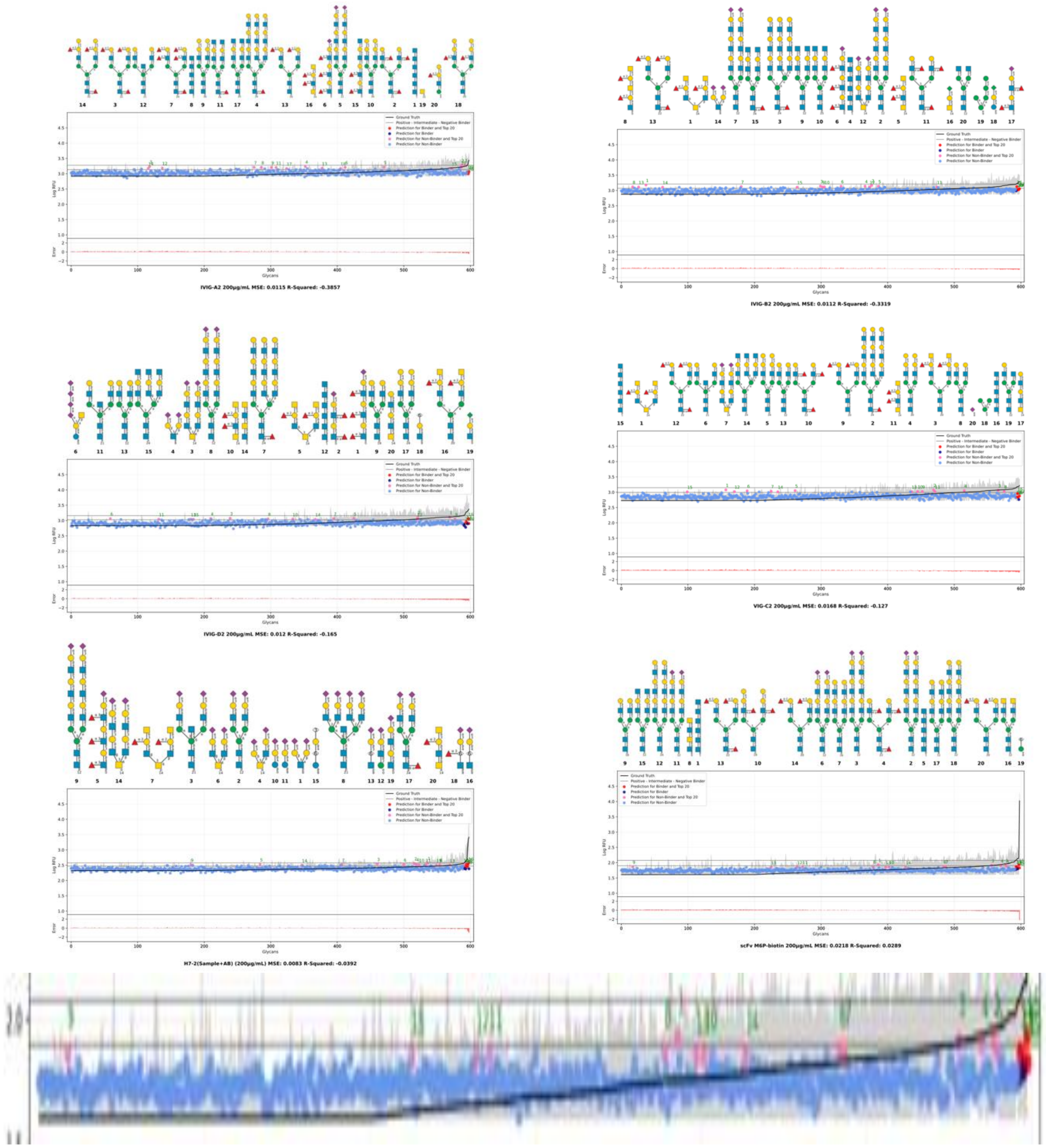

Fig. S5 – Examples of low-MSE-low-R^2 data

Snapshots of six datasets that have low MSE and low R^2. In all six cases, there are no glycans that exhibit RFU signal 10x over the baseline RFU signal and there are few or no glycans that can be classified as “binders”. Zoom in on the data in the bottom right offers some insight: the MSE is low because the absolute difference between ground truth and predicted signal is low. The R^2 is insensitive to the absolute differences, it detects the low correlation between the ground truth and the predicted signal. Still, low R^2 value is inconsequential to predictions in any of these cases because none of these samples contains any binding glycans. Further details can be found in Table S9 - Timestamp Indices for Animations.xlsx

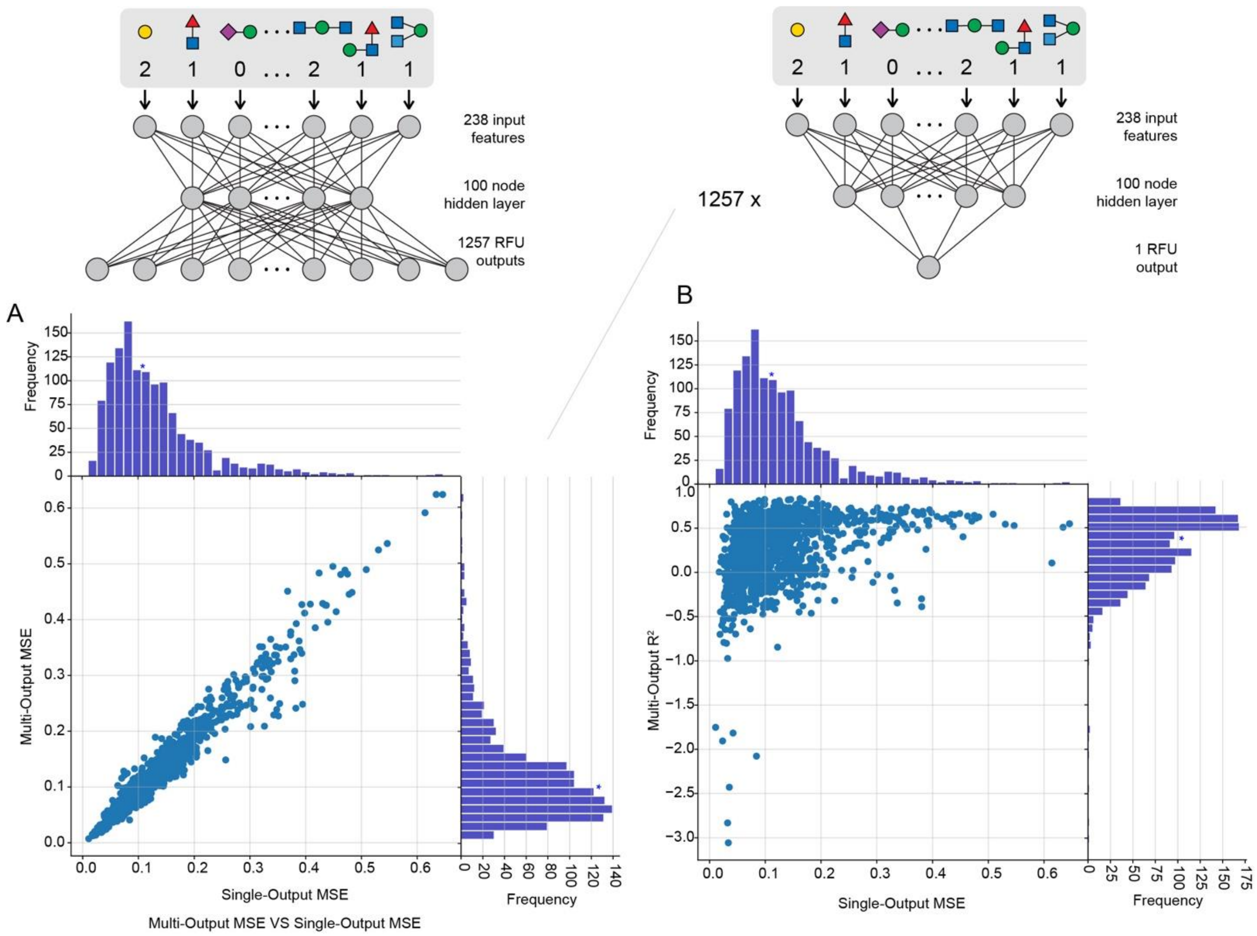

Fig. S6 – Correlation between MSE, R^2 for multi-output vs single output GlyNet

(A) A scatter plot of 1257 MSE values for multi-output GlyNet vs. 1257 single output neural networks (architectures are shown on top). (B) MSE vs. R^2 for 1257 single output neural networks: each dot is prediction for a separate protein sample. The trend of panel B is similar to the trend observed in Figure 3B (similar plot for multi-output GlyNet) but the dynamic range of R^2 is even wider in this case. The same division of glycans into the CV folds was used for the all the single-output and the multi-output networks. We note that the switch to a multi-task network does not benefit all the protein samples, the most extreme decline in MSE being 40% (Supplementary Table S11). The decline appeared to be protein-dependent, for example a list of the negatively impacted cases contained nine samples of Ulex europaeus agglutinin (UEA) and multiple instances of a few other GBPs. This indicates that some aspects of the glycan binding properties of these GBPs was not being properly learned in the multi-output networks, and the single-output networks are able to learn these properties more effectively.

Fig. S7 – The Top 50 Glycans

(*next page*) We used GlyNet model to predict binding 4160 mammalian glycans from the GlyTouCan database to the 1257 protein samples measured using CFG glycan arrays. The images on the next two pages describes the top-50 privileged binding glycan structures that correspond to over 46% of the 10 strongest binders in our predictions. Numbers below the structures are the number of samples for which the glycan is a top-10 binder. The first entry bas been nominated to be a top-10 binder for 505 out of 1257 samples and the next two entries are essentially the same structure, but with fewer repeats. Images are either sorted by the occurrence of glycans (**A**) in the prediction or grouped by similarity (**B**).

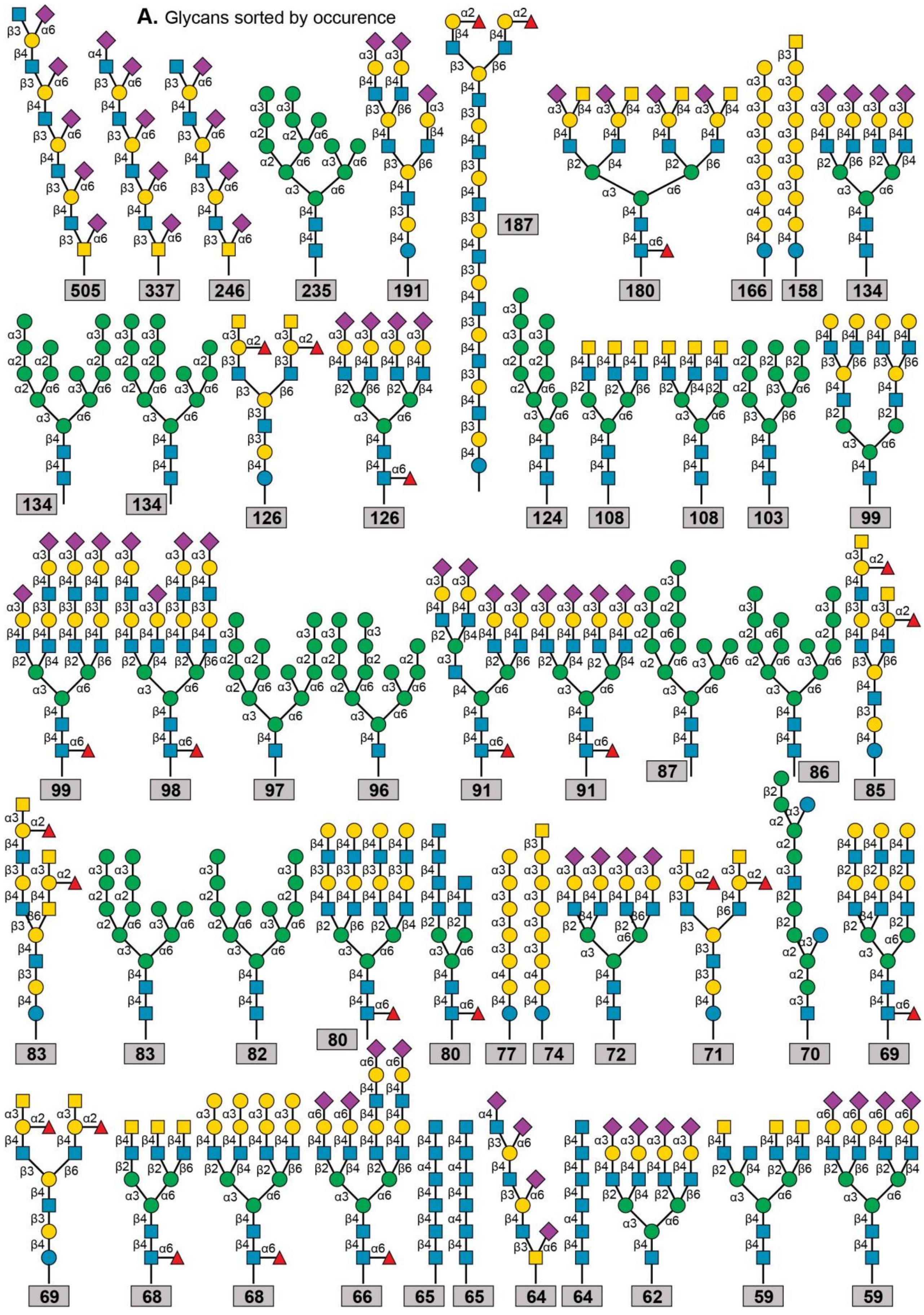
A. Glycans sorted by occurence
505
337
246
235
191
187
180
166
158
134
134
134
126
126
124
108
108
103
99
99
98
97
96
91
91
87
86
85
83
83
82
80
80
77
74
72
71
70
69
69
68
68
66
65
65
64
64
62
59
59

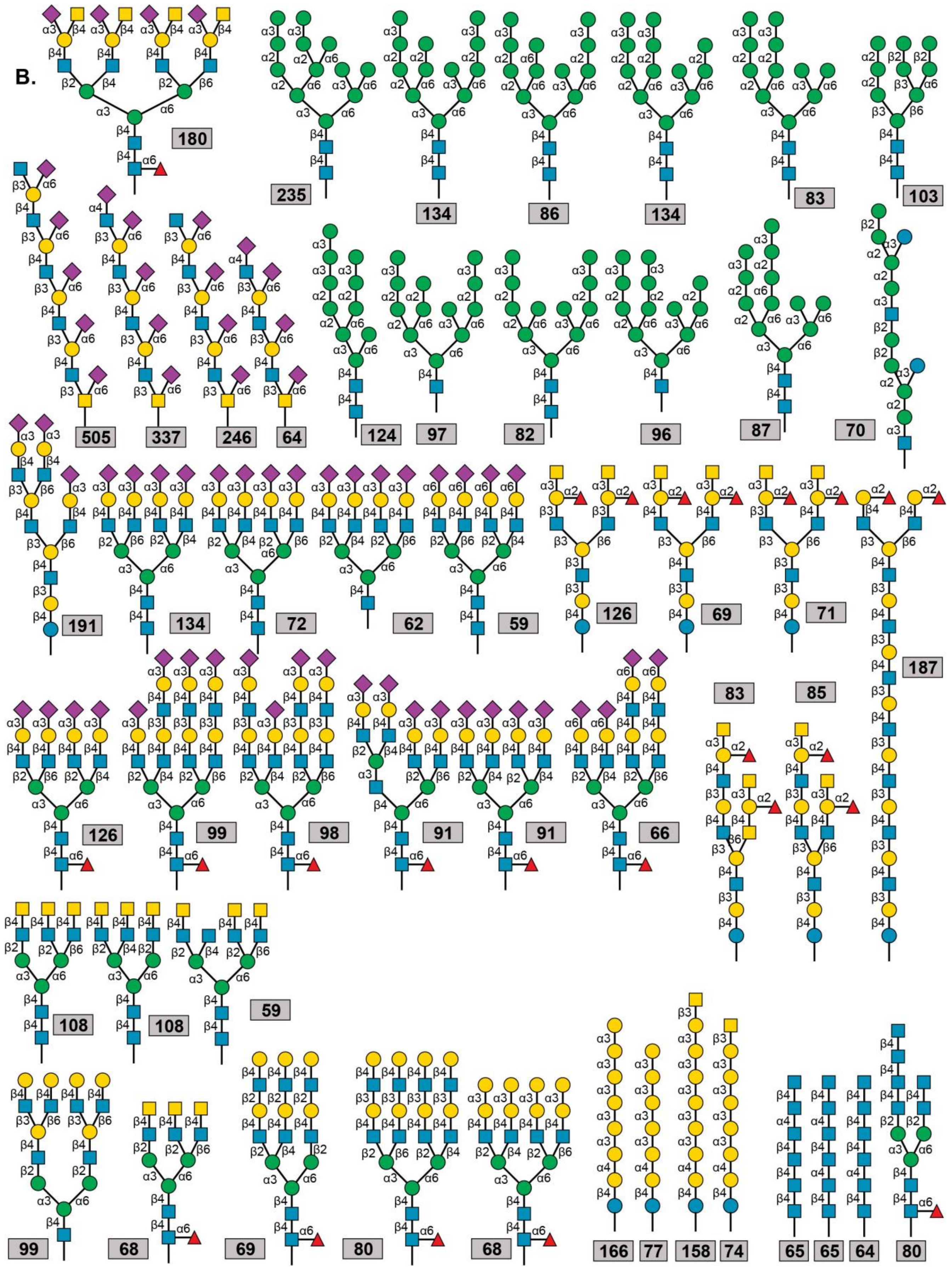
B.
180
235
134
86
134
83
103
505
337
246
64
124
97
82
96
87
70
191
134
72
62
59
126
69
71
187
83
85
126
99
98
91
91
66
108
108
59
99
68
69
80
68
166
77
158
74
65
65
64
80

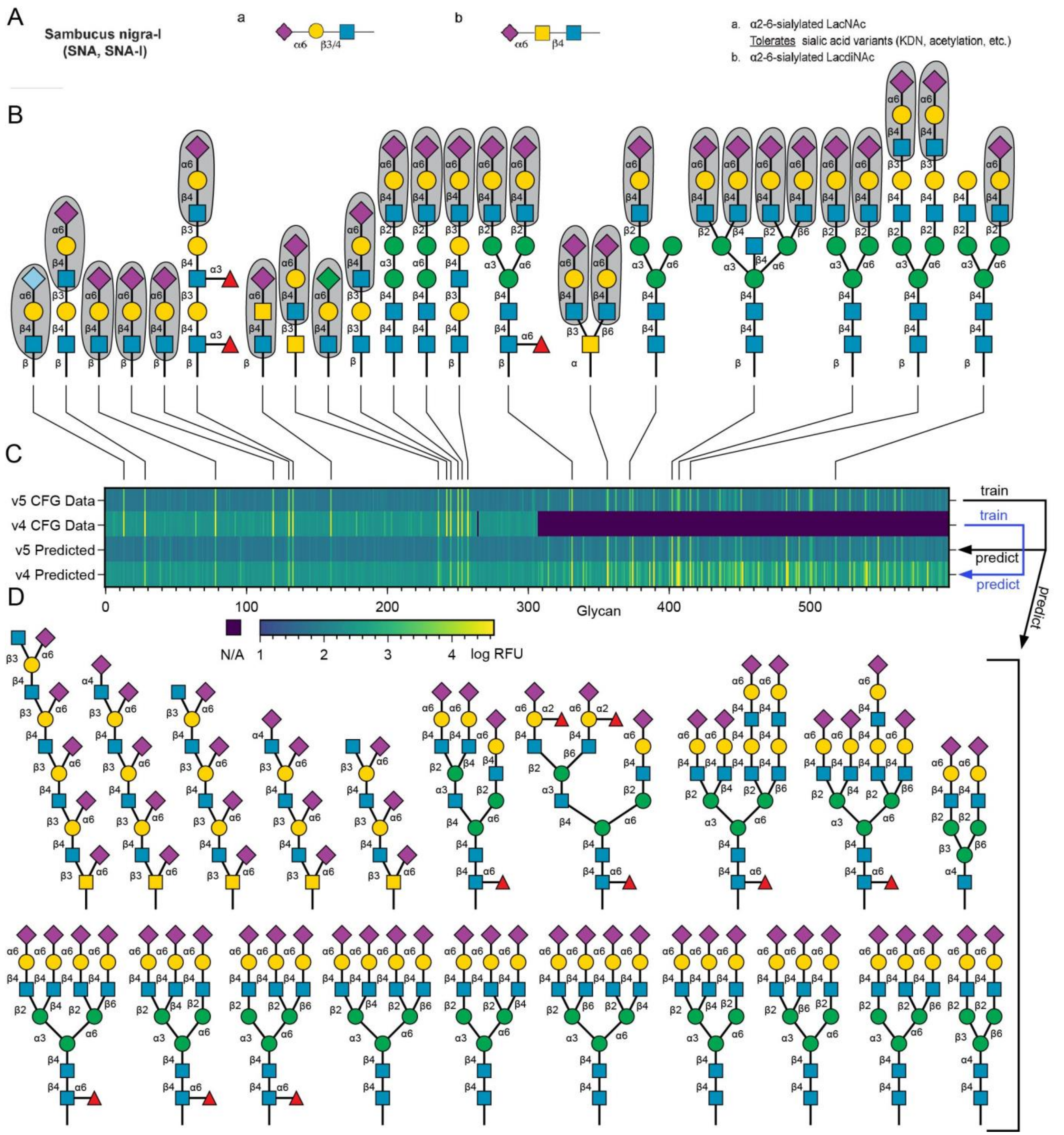

Fig. S8 – Glycan Binding to SNA

A) SNA binding motif reported by Mahal and coworkers[5], (figure copied from Ref[5]). B) Representative glycan structures with strong binding to SNA in the CFG data. C) Heatmap of binding of the 599 glycans to SNA samples. The top two rows are experimentally measured CFG log-RFUs: **Row 1:** 599 glycans from a v5 array (1 μg/mL SNA; ID: 1004702); **Row 2:** 293 matching glycans from a v4 array (also 1 μg/mL SNA; ID: 1004421). The bottom two rows are GlyNet predictions made in hold-out folds: **Row 3:** outputs trained to match the v5 array; **Row 4:** outputs trained to match the v4 array glycans and prediction for the 306 glycans not available in v4 arrays. D) The 20 glycans from the 4160 novel glycans from the GlyTouCan database predicted by GlyNet to have the highest binding to the 1 μg/mL SNA sample. All 20 predicted glycans contain canonical SNA binding motifs.

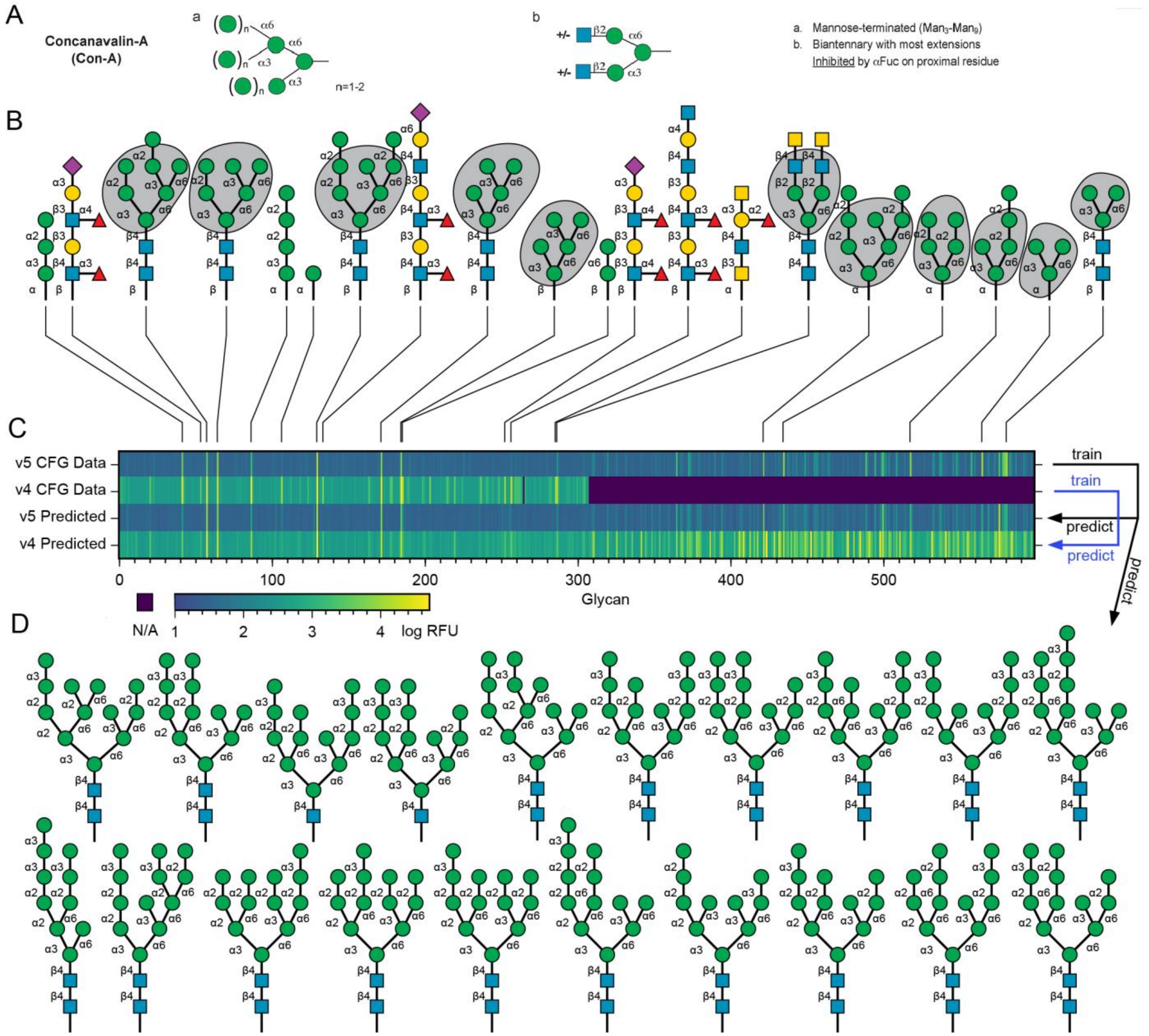

Fig. S9 – Glycan Binding to ConA

A) ConA binding motif reported by Mahal and coworkers[5], (figure copied from Ref[5]). B) Representative glycan structures with strong binding to ConA in the CFG data. C) Heatmap of binding of the 599 glycans to ConA samples. The top two rows are experimentally measured CFG log-RFUs: **Row 1:** 599 glycans from a v5 array (1 µg/mL ConA; ID: 1004465); **Row 2:** 293 matching glycans from a v4 array (1 µg/mL ConA; ID: 1004412). The bottom two rows are GlyNet predictions made in hold-out folds: **Row 3:** outputs trained to match the version 5 array; **Row 4:** outputs trained to match the v4 array data and predictions for 306 glycans not available in v4 arrays. D) The 20 glycans from the 4160 novel glycans from GlyTouCan database predicted by GlyNet to have the highest binding to 1 µg/mL ConA sample (CFG ID: 1004455). All 20 of these glycans contain canonical ConA binding motifs.

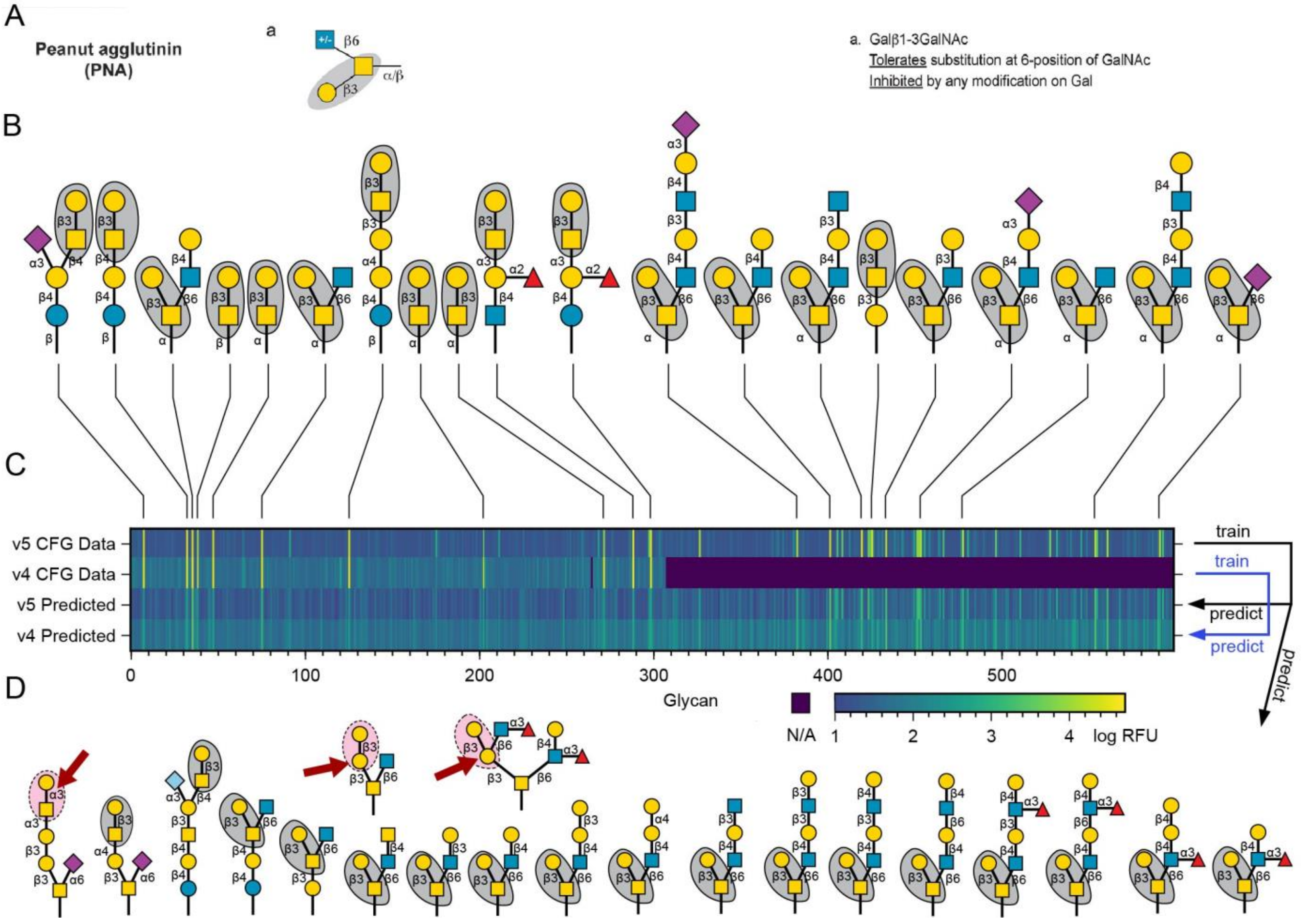

Fig. S10 – Glycan Binding to PNA

A) The lectin binding motif reported by Mahal and coworkers[5], (figure copied from Ref[5]).

B) Representative glycan structures with strong binding to PNA in the CFG data. C) Heatmap of binding of the 599 glycans to PNA samples. The top two rows are experimentally measured CFG log-RFUs: **Row 1**: 599 glycans from a v5 array (10 µg/mL PNA; ID: 1004677); **Row 2**: 293 matching glycans from a v4 array (10 µg/mL PNA; ID: 1004375). The bottom two rows are GlyNet predictions made in hold-out folds: **Row 3**: outputs trained to match the v5 array; **Row 4**: outputs trained to match the v4 array glycans and new prediction for 306 glycans not available in v4 arrays. The model attenuates the intensity of the binding, but it effectively predicts the glycans that have the strongest interactions with PNA. D) The 20 glycans from the 4160 novel glycans from GlyTouCan database predicted by GlyNet to have the highest binding to 1 µg/mL ConA sample (CFG ID: 1004667). Of the 20 predicted strongly-binding glycans, 17 contain canonical PNA binding motifs and three glycans contain close variants of the canonical motif (β3-to-α3 change in the stereochemistry or GalNAc-to-Gal change as indicated by a crimson arrow).

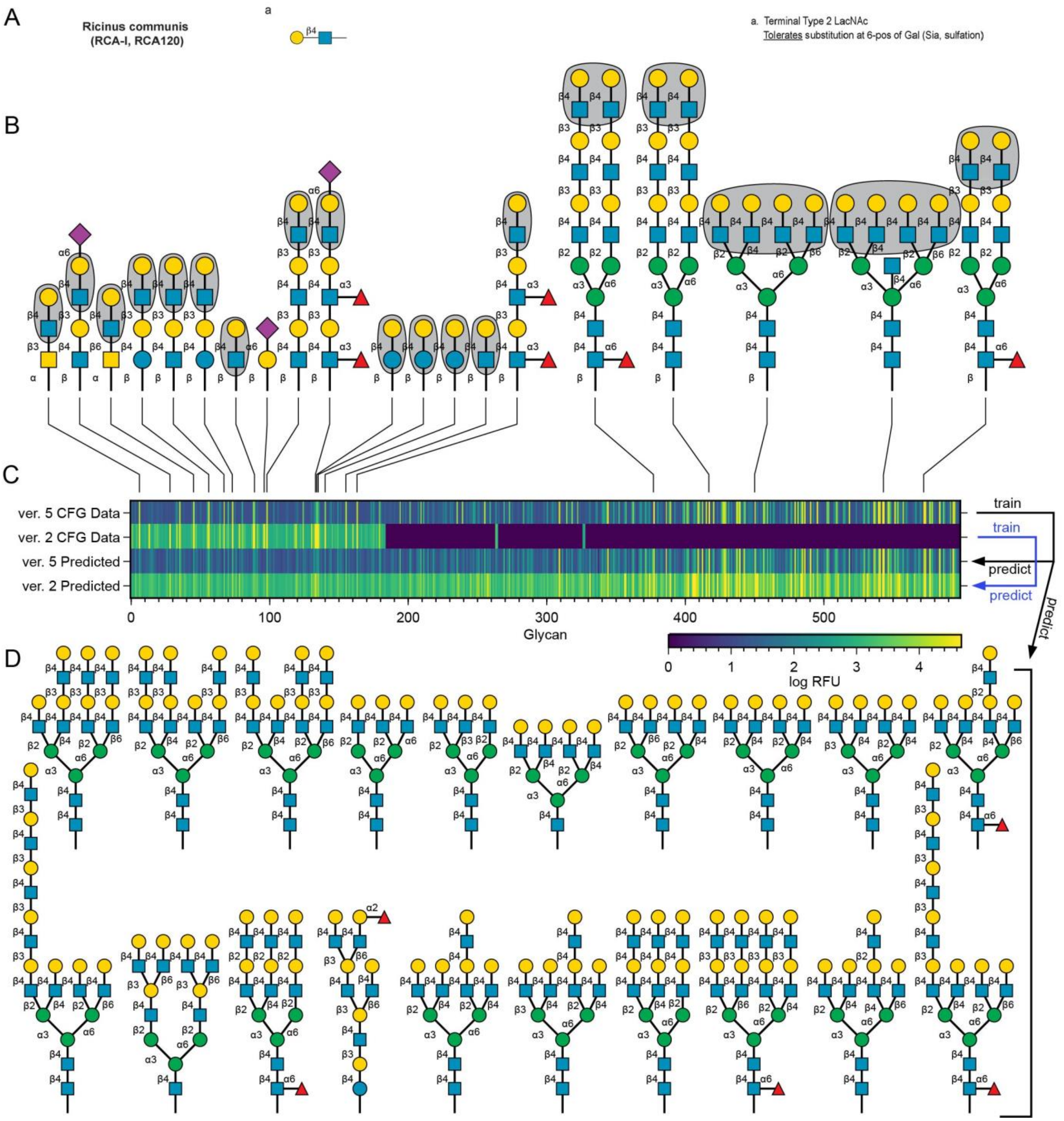

Fig. S11 – Glycan Binding to RCA

A) RCA binding motif reported by Mahal and coworkers[5], (figure copied from Ref[5]). B) Representative glycan structures with strong binding to RCA in the CFG data. C) Heatmap of binding of the 599 glycans to RCA samples. **Row 1**: experimentally measured CFG log-RFUs from 599 glycans from a v5 array (1 µg/mL RCA; ID 10044659); **Row 2**: measured CFG log-RFUs from 188 matching glycans from a v2 array (2 µg/mL RCA; ID 10044600). Rows 3-4 are GlyNet predictions made in hold-out folds: **Row 3**: outputs trained to reproduce the v5 array; **Row 4**: output predictions for the v2 array glycans and predictions for 411 glycans not available in v2 arrays. Despite the elevated baseline in the v2 training set (**row 2**) and 411/599=69% of the binding data missing from v2 dataset when compared to the v5 dataset, the extrapolated predictions for the v2 dataset (**row 4**) have strong binding to a majority

of the RCA-binding glycans experimentally validated in the v5 set (**row 1**). D) The 20 glycans from the 4160 novel glycans from the GlyTouCan database predicted by GlyNet to have the highest binding to the 1 µg/mL RCA sample (CFG ID: 10044659). All 20 predicted glycans contain 3-4 copies of the canonical RCA binding motif: terminal Galβ(1−4)GlcNAc.

Legend for the figure on the next page:

Fig. S12 – Glycan Binding to UEA I
A) The canonical UEA I binding motifs reported by Mahal and coworkers[5], (figure from Ref[5]). B) Representative glycan structures with strong binding to UEA I in the CFG data. C) Heatmap of binding for the 599 glycans to UEA I samples. Rows 1-14 are experimentally measured log-RFUs (see legend of ESI Fig. S13 for primscreen ID and more details of individual samples).
Rows 1-3: 599 glycans from v5 arrays (0.1, 1, 100 µg/mL UEA I from Vector Laboratories);
Row 4: 599 glycans from v5 arrays (10 µg/mL UEA I from EY Laboratories, Inc.);
Rows 5-11: 599 glycans from v5 arrays (0.01, 0.1, 0.5, 1, 10, 50, 100 µg/mL UEA I from EY Labs);
Rows 12-14: 293 matching glycans from v4 arrays (0.3, 3, 30 µg/mL UEA I, source unknown).
Red lines highlight binding of non-canonical (β4-GalNAc-)-oligomers (chitin oligomers) and other non-canonical motifs detailed in ESI Fig. S13 to the v5 arrays probed with UEA I from EY Labs but not the v4 arrays probed by UEA I from Vector Labs. We hypothesize that these differences are the result of differences in the source, extraction/purification or perhaps even contamination of one of the batches of UEA I (see ESI Fig. S13 for a more detailed analysis of the differences).

Rows 15-28 are GlyNet predictions made in hold-out folds:
Rows 1-3: predictions trained to match v5 array data (0.1, 1, 100 µg/mL UEA I from Vector Labs);
Row 4: predictions trained to match v5 array data (10 µg/mL UEA I from EY Labs);
Rows 5-11: predictions matching v5 data (0.01, 0.1, 0.5, 1, 10, 50, 100 µg/mL UEA I from EY Labs).
Row 12-14: data trained to match v4 array data (0.3, 3, 30 µg/mL UEA I from unknown source) and predictions for 306 glycans not in the v4 arrays.
The GlyNet predictions are attenuated in binding intensity, but they recapitulate the pattern of binding to canonical motifs. UEA I exhibited a significant divergence in experimental binding depending on the protein source. For example, non-canonical binding to (β4-GalNAc-)-oligomers is pronounced in UEA I from EY Laboratories, but missing in UEA I from Vector Laboratories and in UEA I samples run on v4 arrays. As a result, the predictions also diverge; specifically, held-out predictions for the EY Laboratories sourced UEA I have strong binding to (β4-GalNAc-)-penta- and hexasacharides matching the experimental observations, but for both UEA from Vector Laboratories and samples run on v4 arrays the held-out predictions for (β4-GalNAc-) exhibit no binding.
D) The 20 glycans from the 4160 novel GlyTouCan glycans predicted by GlyNet to have the highest binding to the 50 µg/mL EY Lab UEA I sample. Of the 20, 18 contain canonical UEA binding motifs, whereas two possess the (β4-GalNAc-)-oligomer binding motif, in accordance with these non-canonical motifs being observed to bind strongly in the corresponding experimental data.

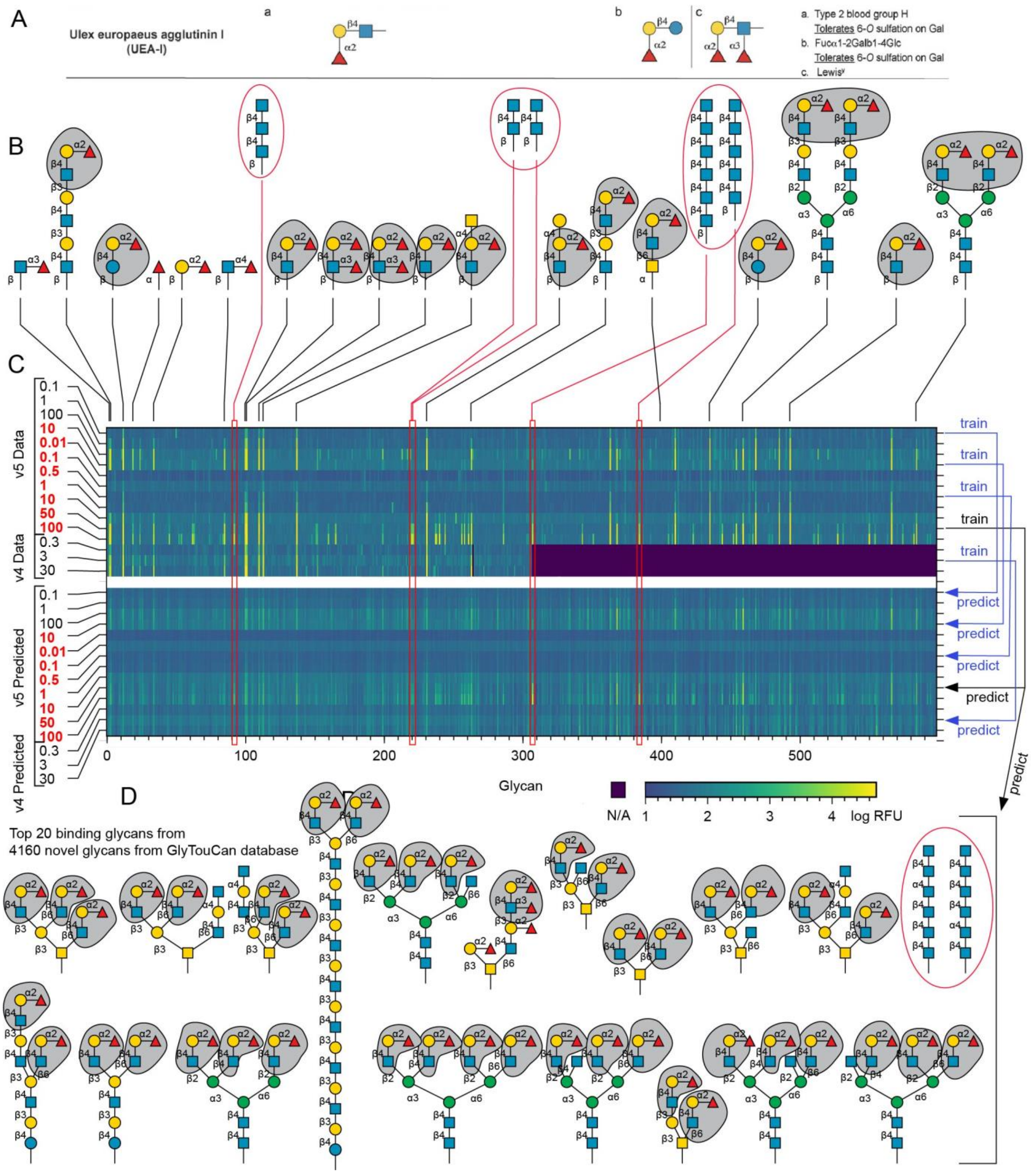

A
Ulex europaeus agglutinin I
(UEA-I)
a. Type 2 blood group H
Tolerates 6-O sulfation on Gal
b. Fucα1-2Galb1-4Glc
Tolerates 6-O sulfation on Gal
c. Lewis^y
B
C
v5 Data
v4 Data
v5 Predicted
v4 Predicted
train
predict
Glycan
N/A
log RFU
D
Top 20 binding glycans from
4160 novel glycans from GlyTouCan database

Legend for the figure on the next page:

Fig. S13 – Patterns of Glycan Binding to UEA I in the CFG Data
Detailed examination of canonical and non-canonical binding preferences of UEA I lectin based on an extract of ESI Table S4, which gives log-scaled RFU values for several protein samples across v5 arrays. Here we have extracted samples from multiple sources of UEA I applied at a range of concentrations. These have been supplemented with data from three samples run on v4 arrays, missing values are glycans not present in these arrays. The first three columns were measured using UEA I for which we do not know the source, whereas a vendor is known for the other cases; eight columns were acquired using Biotin-UEA-I from EY Laboratories, and the last three columns were measured using UEA I from Vector Laboratories. The strongest interactions are coloured yellow, with green for intermediate values, and with blue for the weakest interactions. Besides the binding motif reported by Mahal and coworkers (A)[5] Biotin-UEA-I from EY Labs at 50-100 µg/mL binds to at least three other glycan patterns: B) GlcNAc(β1-4)GlcNAc also seen in ESI Figs. S12 and S14, C) GlcNAc(α1-3/4)Gal(β1-4)GlcNAc, and D) GalNAc(β1-4)GlcNAc. The structures in B and D seem to tolerate modification by sulfate or sialic acid in the 6-position but not in positions 3 or 4.

All measurements in Figure S13 are from the following CFG samples:

UEA I protein: Unknown source CFG: cbp 1010

1. 1004420 primscreen_4421 0.3 µg/mL
2. 1004419 primscreen_4420 3 µg/mL
3. 1004418 primscreen_4419 30 µg/mL

UEA I protein: EY Labs Cat. #: BA-2201-2 Lot #: 290224-2 CFG cbp 2532

4. 1004737 primscreen_4738 10 µg/mL

UEA I protein: EY Labs Cat. #: BA-2201-2 Lot #: 290224-1 CFG cbp 2787

5. 1004796 primscreen_4797 0.01 µg/mL
6. 1004797 primscreen_4798 0.1 µg/mL
7. 1004798 primscreen_4799 0.5 µg/mL
8. 1004799 primscreen_4800 1 µg/mL
9. 1004802 primscreen_4803 10 µg/mL
10. 1004800 primscreen_4801 50 µg/mL
11. 1004801 primscreen_4802 100 µg/mL

UEA I protein: Vector Labs Cat. #: B-1065 Lot #: V 1207 CFG cbp 2481

12. 1004733 primscreen_4734 0.1 µg/mL
13. 1004734 primscreen_4735 1 µg/mL
14. 1004736 primscreen_4737 100 µg/mL

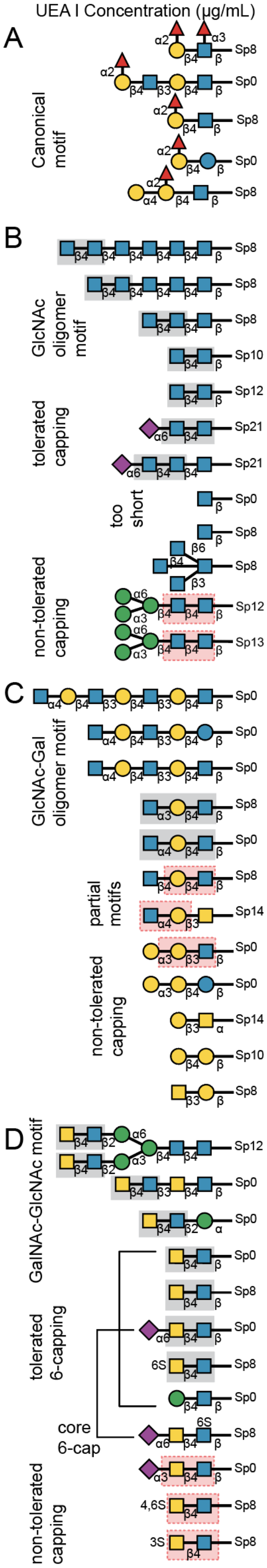

	UEA I, unknown source			UEA I, EY Lab								UEA I, Vector Lab			
CFG Array Sample ID	1004420	1004419	1004418	1004737	1004796	1004797	1004798	1004799	1004802	1004800	1004801	1004733	1004734	1004736	GlyTouCan Accession
UEA I Concentration (μg/mL)	0.3	3	30	10	0.01	0.1	0.5	1	10	50	100	0.1	1	100	
A	3.6	4.3	4.4	4.5	1.3	1.8	2.5	2.8	4.4	4.6	4.4	2.8	3.8	4.4	G00052MO
A	3.1	3.9	4.4	4.0	1.4	1.6	1.7	2.0	3.8	4.4	4.3	1.7	2.9	4.3	G29703NT
A	4.3	4.5	4.5	4.6	1.9	2.7	3.5	3.8	4.5	4.6	4.6	3.3	4.2	4.5	G61459JW
A	4.1	4.4	4.5	4.5	1.7	2.6	3.1	3.4	4.3	4.5	4.5	3.3	4.0	4.5	G61399NC
A	4.1	4.5	4.5	4.6	1.5	2.8	2.6	3.7	4.7	4.5	4.5	3.1	4.2	4.5	G46849JR
B				3.1	1.3	1.7	2.0	2.1	3.3	4.1	4.4	1.6	1.7	2.3	G36414FS
B				2.6	1.6	1.7	1.6	1.8	3.1	4.0	4.4	1.6	1.6	1.7	G07670VS
B	2.7	1.6	1.5	2.5	1.4	1.6	1.6	1.7	2.9	3.7	4.5	1.5	1.6	1.8	G47362BJ
B	1.5	1.7	1.5	2.0	1.4	1.6	1.4	1.5	2.3	3.5	4.5	1.6	1.5	1.8	G42666HT
B	1.7	2.0	1.6	1.9	1.6	1.7	1.5	1.6	1.8	3.5	4.1	1.8	1.5	1.7	G42666HT
B	1.5	2.2	1.5	1.8	1.3	1.6	1.4	1.4	1.9	2.8	4.1	1.5	1.5	1.7	G25824CS
B	1.7	2.0	2.5	2.1	1.3	1.6	1.4	1.5	2.3	3.4	4.5	1.6	1.7	1.7	G29489NE
B	1.5	1.6	1.6	1.9	1.6	1.6	1.5	1.7	1.8	1.7	1.8	1.5	1.6	1.7	G49108TO
B	1.6	1.6	1.6	1.9	1.5	1.6	1.4	1.6	1.8	1.6	1.7	1.5	1.5	1.8	G49108TO
B				1.9	1.4	1.7	1.4	1.4	1.8	1.7	1.7	1.6	1.5	1.7	G73646HZ
B				1.9	1.6	1.7	1.5	1.4	1.8	1.8	1.7	1.5	1.6	1.7	G22768VO
B				1.9	1.4	1.8	1.5	1.5	1.8	1.7	2.0	1.5	1.8	1.8	G22768VO
C	1.7	1.9	1.6	2.6	1.5	1.6	1.7	1.5	2.9	4.1	4.5	1.7	1.9	1.9	G70021TR
C	1.5	1.9	1.5	2.1	1.4	1.6	1.4	1.5	2.7	3.8	4.5	1.6	1.5	1.8	G20818VS
C	1.5	1.7	1.6	2.0	1.3	1.6	1.4	1.6	2.8	3.4	4.4	1.6	1.5	1.7	G32357ON
C	1.5	1.8	1.5	2.5	1.3	1.6	1.4	1.5	2.1	3.6	4.2	1.6	1.5	1.8	G66916RU
C	3.0	1.8	1.7	2.1	1.3	1.6	1.4	1.7	2.9	3.8	4.5	1.6	1.5	1.7	G07397MN
C	1.5	1.7	1.5	1.8	1.3	1.9	1.4	1.6	1.8	1.6	1.7	1.5	1.6	1.7	G51131QL
C	1.5	2.0	1.6	1.9	1.4	1.7	1.4	1.5	1.8	1.6	1.7	1.8	1.8	1.7	G64354IV
C	1.5	1.6	1.7	1.8	1.4	1.7	1.4	1.5	1.8	1.6	1.8	1.5	1.5	1.8	G34356AA
C	1.5	1.6	1.7	1.9	1.3	1.7	1.4	1.4	1.7	1.6	1.7	1.5	1.5	1.9	G78074HX
C	1.5	1.8	1.6	1.8	1.6	1.7	1.5	1.4	1.8	1.7	1.7	1.6	1.7	1.9	G00031MO
C	1.5	1.7	1.6	1.9	1.4	1.6	1.4	1.4	1.8	1.6	1.7	1.5	1.5	1.9	G67272YR
C	1.6	1.7	1.6	1.8	1.3	1.7	1.4	1.4	1.7	1.6	1.7	1.6	1.6	1.8	G08806VI
D	1.8	1.7	1.7	2.5	2.2	2.3	2.4	2.4	2.4	3.3	3.8	2.0	1.9	2.1	G83062JW
D				1.9	1.3	1.6	1.4	1.5	1.8	3.2	4.2	1.5	1.6	1.7	G88274ER
D				2.3	1.3	1.6	1.4	1.5	2.6	3.3	4.6	1.6	1.8	1.7	G71793UG
D	1.6	2.0	1.5	1.9	1.3	1.6	1.4	1.5	1.7	3.0	3.6	1.5	1.6	1.7	G00057MO
D	1.5	1.7	1.8	2.4	1.3	1.6	1.5	1.5	2.1	3.2	4.2	1.5	1.5	1.7	G00057MO
D	1.5	1.6	1.5	2.0	1.3	1.7	1.5	1.4	1.8	2.7	3.8	1.5	1.5	2.6	G54118CT
D				1.9	1.3	1.6	1.4	1.4	2.2	3.3	4.3	1.5	1.6	1.8	G76572KX
D	1.5	1.9	1.6	1.8	1.5	1.7	1.4	1.7	1.8	1.6	1.7	1.5	1.6	1.7	G90333CG
D				1.8	1.3	1.6	1.4	1.4	1.7	1.6	1.7	1.5	1.5	1.7	G02967YO
D	1.6	1.8	1.5	1.8	1.3	1.6	1.4	1.5	1.7	1.6	1.8	1.5	1.5	1.7	G95440AP
D				1.8	1.3	1.6	1.4	1.4	1.7	1.6	1.7	1.5	1.6	1.7	G07027EE
D				1.9	1.5	1.6	1.4	1.4	1.7	1.6	1.7	1.5	1.5	1.7	G87277AB

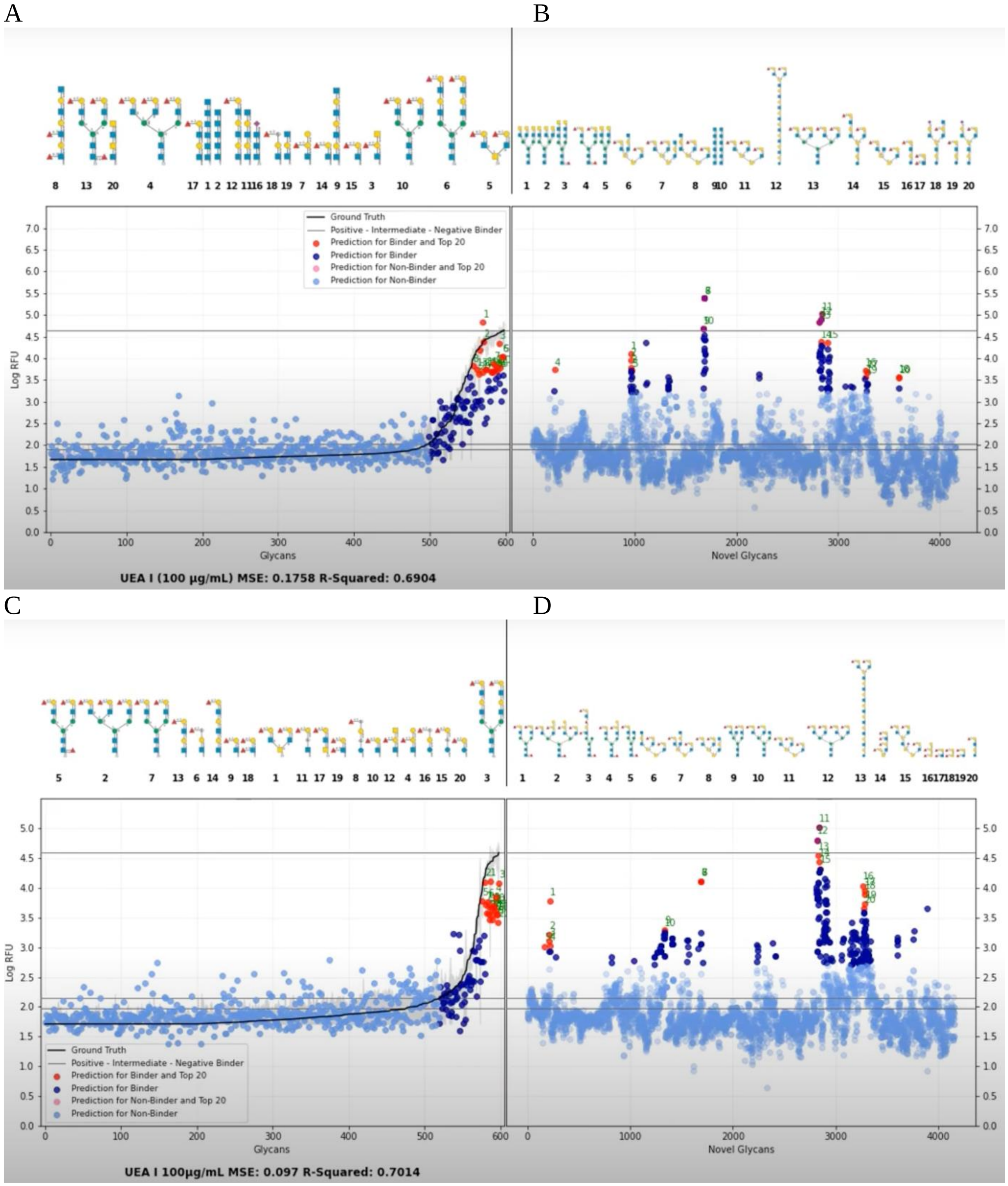

Fig. S14 –Glycan Binding to UEA I
(**A**) Snapshots taken from https://youtu.be/pa_6nO0Zl64?t=1156 (frame at 1156 seconds) detailing the binding of EY Labs UEA I lectin at 100 µg/mL to CFG ver 5 array and (B) predictions made by GlyNet for the ability of this EY Laboratories UEA I to bind 4160 glycans from GlyTouCan database. (**C**) Snapshots taken from https://youtu.be/pa_6nO0Zl64?t=1165 (frame at 1165 seconds) detailing the binding of Vector Labs UEA I lectin at 100 µg/mL to CFG ver 5 array and (**D**) predictions made by GlyNet for the ability of this Vector Laboratories UEA I to bind 4160 glycans from GlyTouCan database. In panel (**A**), glycans **1** and **2** (β4-GalNAc-oligomers) do not contain a canonical UEA I recognition motif but they are among the strongest binding experimentally measured binders for EY

Laboratories UEA I at 100 µg/mL; In panel (**B**), the predicted binders to EY UEA I at 100 µg/mL also contain non-canonical (β4-GalNAc-)-oligomer motifs (glycans **9** and **10**). In contrast non-canonical glycan binders are not observed experimentally in binding to Vector Laboratories UEA I. Predicted binders from GlyTouCan database also do not contain non-canonical UEA I motifs (**D**). Overall, there is a divergence in predicted binding glycans for EY Laboratories (**B**) and Vector Laboratories proteins (**D**).

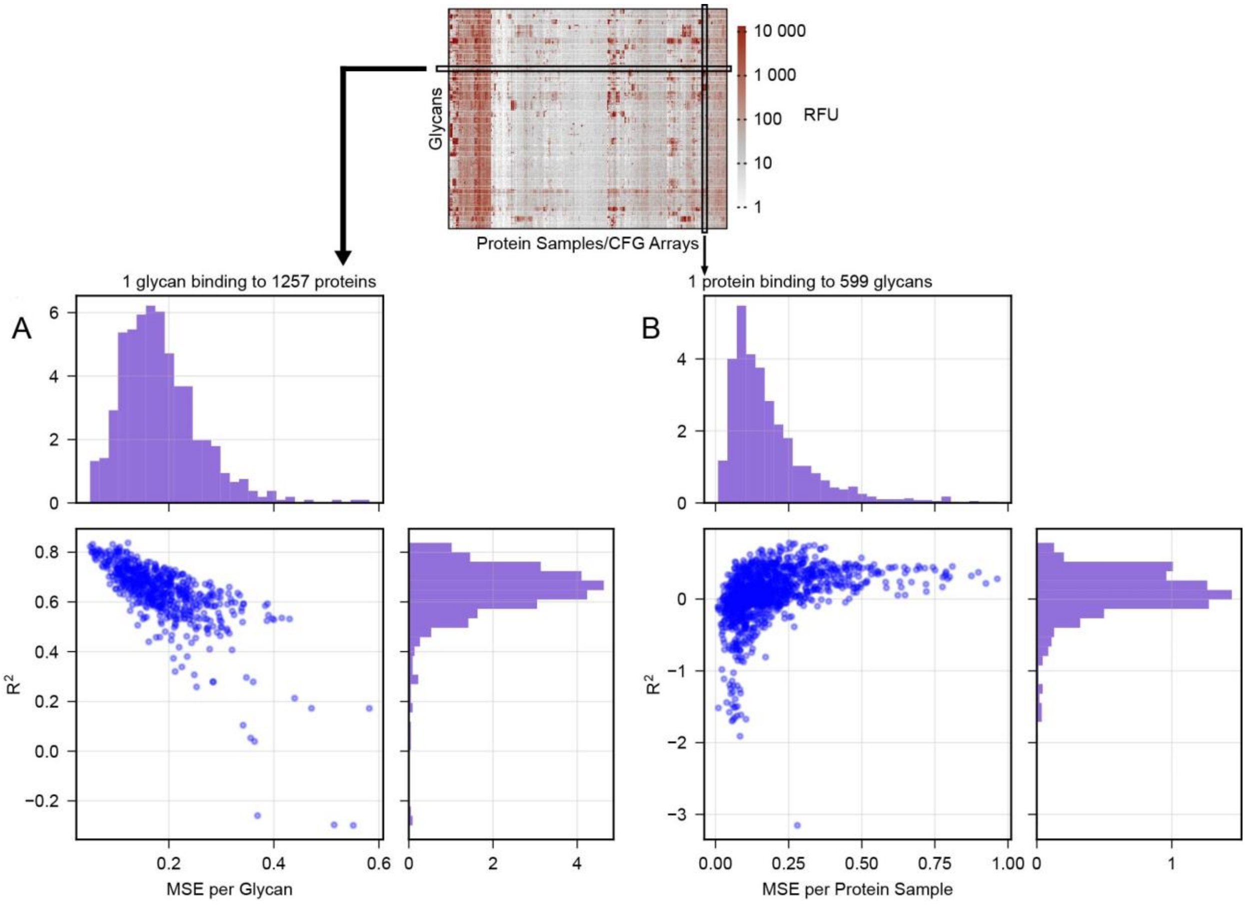

Fig. S15 – Analysis of Predictions from the SweetNet Multi-output Models

Analysis in the style of Fig. S3 of relation between MSE and R^2 by A) protein samples and B) glycan on hold-out fold predictions from the multi-output SweetNet[4] models. Observations on this figure parallel those of our data in Fig. S3.

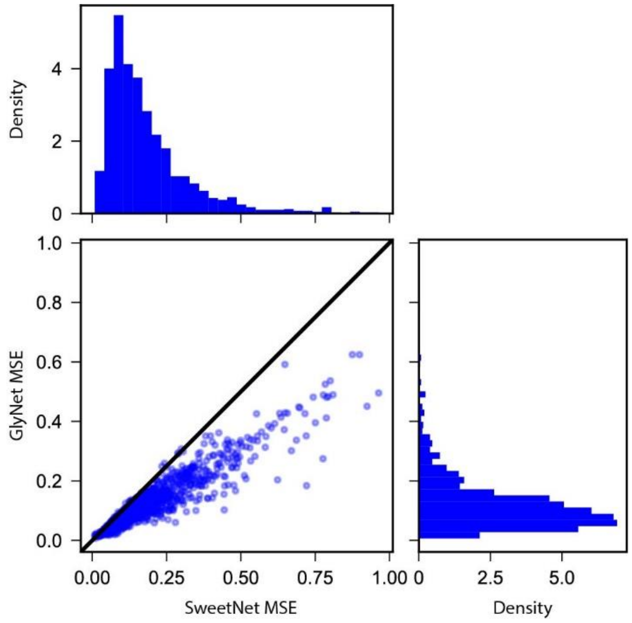

3.1 *Fig. S16 – Comparison of MSE Distribution between the SweetNet and GlyNet Models*

The per protein sample MSEs from hold out fold predictions produced by both systems are plotted. The correlation between the MSEs indicates that in general both architectures perform relatively well or poorly on particular samples.

4 References

1. Coff, L., Chan, J., Ramsland, P. A. & Guy, A. J. Identifying glycan motifs using a novel subtree mining approach. *BMC Bioinformatics* **21**, 42, doi:10.1186/s12859-020-3374-4 (2020).
2. Paszke, A. *et al.* PyTorch: An Imperative Style, High-Performance Deep Learning Library. *Adv. Neur. In.* **32**, 8024-8035 (2019).
3. Kingma, D. P. & Ba, J. Adam: A Method for Stochastic Optimization. *Proceedings of the 3rd International Conference on Learning Representations (ICLR)* (2014).
4. Burkholz, R., Quackenbush, J. & Bojar, D. Using graph convolutional neural networks to learn a representation for glycans. *Cell Rep* **35**, 109251, doi:10.1016/j.celrep.2021.109251 (2021).
5. Bojar, D. *et al.* A Useful Guide to Lectin Binding: Machine-Learning Directed Annotation of 57 Unique Lectin Specificities. *ACS Chem Biol*, doi:10.1021/acschembio.1c00689 (2022).
