# Supplementary figures and images for: GlyNet: a multi-task neural network for predicting protein–glycan interactions
Source: Chem Sci. 2022 May 16;13(22):6669–86. doi: 10.1039/d1sc05681f (PMC9172296; doi:10.1039/d1sc05681f)

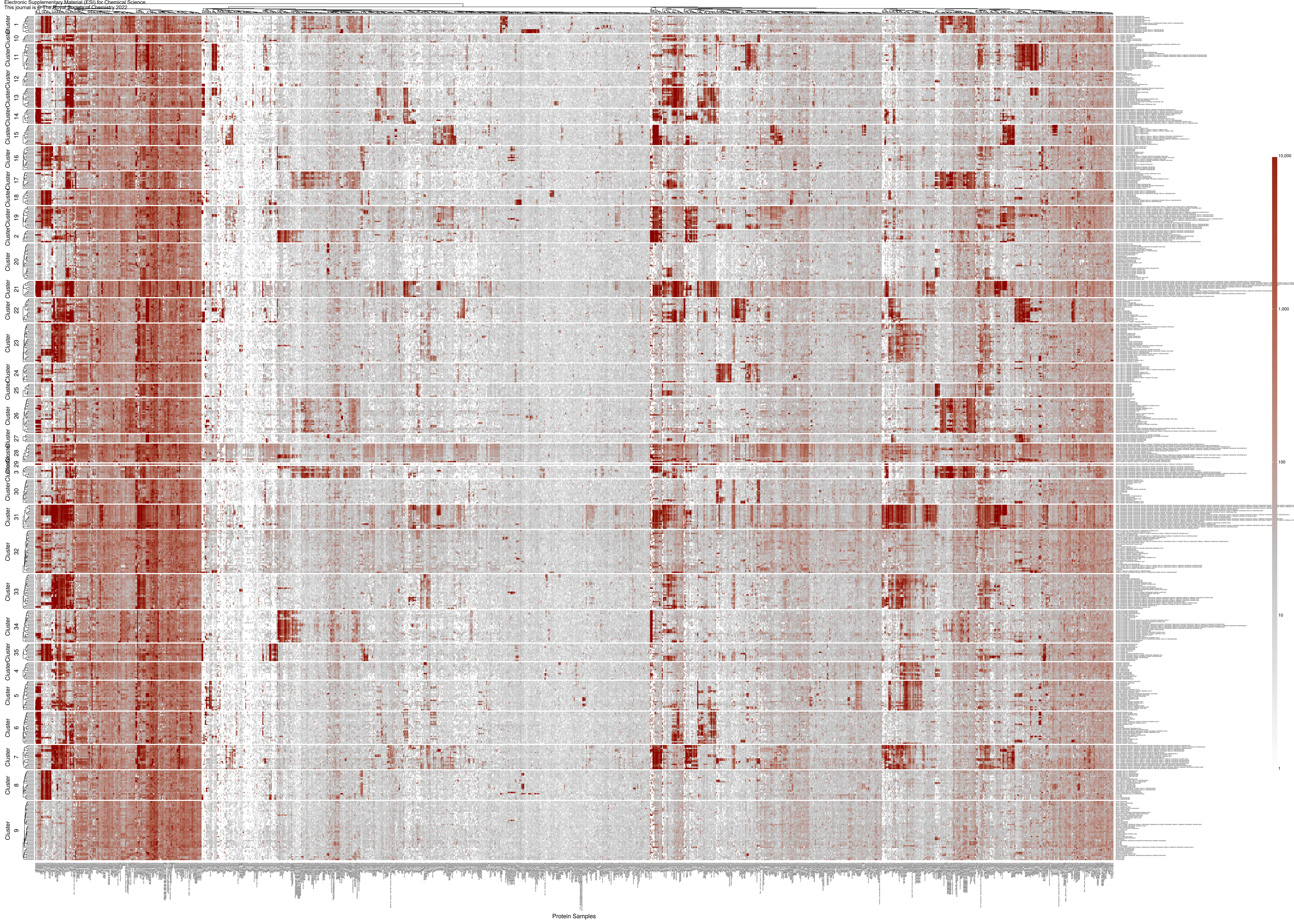

Supplement: SC-013-D1SC05681F-s002 [file SC-013-D1SC05681F-s002.pdf]
